# The geometry of reaction norms yields insights on classical fitness functions for Great Lakes salmon

**DOI:** 10.1371/journal.pone.0228990

**Published:** 2020-03-16

**Authors:** James E. Breck, Carl P. Simon, Edward S. Rutherford, Bobbi S. Low, P. J. Lamberson, Mark W. Rogers

**Affiliations:** 1 School for Environment and Sustainability, The University of Michigan, Ann Arbor, Michigan, United States of America; 2 Center for the Study of Complex Systems, The University of Michigan, Ann Arbor, Michigan, United States of America; 3 Department of Mathematics, The University of Michigan, Ann Arbor, Michigan, United States of America; 4 Gerald R. Ford School of Public Policy, The University of Michigan, Ann Arbor, Michigan, United States of America; 5 National Oceanic and Atmospheric Administration Great Lakes Environmental Research Laboratory, Ann Arbor, Michigan, United States of America; 6 Department of Communication, University of California–Los Angeles, Los Angeles, California, United States of America; 7 U.S. Geological Survey, Tennessee Cooperative Fishery Research Unit, Tennessee Technological University, Cookeville, Tennessee, United States of America; University of Maine at Farmington, UNITED STATES

## Abstract

Life history theory examines how characteristics of organisms, such as age and size at maturity, may vary through natural selection as evolutionary responses that optimize fitness. Here we ask how predictions of age and size at maturity differ for the three classical fitness functions–intrinsic rate of natural increase *r*, net reproductive rate *R*_0_, and reproductive value *V*_*x*_−for semelparous species. We show that different choices of fitness functions can lead to very different predictions of species behavior. In one’s efforts to understand an organism’s behavior and to develop effective conservation and management policies, the choice of fitness function matters. The central ingredient of our approach is the maturation reaction norm (MRN), which describes how optimal age and size at maturation vary with growth rate or mortality rate. We develop a practical geometric construction of MRNs that allows us to include different growth functions (linear growth and nonlinear von Bertalanffy growth in length) and develop two-dimensional MRNs useful for quantifying growth-mortality trade-offs. We relate our approach to Beverton-Holt life history invariants and to the Stearns-Koella categorization of MRNs. We conclude with a detailed discussion of life history parameters for Great Lakes Chinook Salmon and demonstrate that age and size at maturity are consistent with predictions using *R*_0_ (but not *r* or *V*_*x*_) as the underlying fitness function.

## Introduction

Life history theory concerns the type, timing, and duration of important events in an organism’s lifetime, including birth, weaning, dispersal, sexual maturity, mating, caring for young, and senescence [1,2]. Knowledge of these life history parameters is an indispensable ingredient for understanding organism behavior and for designing policies on stocking, harvesting, and conservation in general. This study was catalyzed by the observation that different populations of salmonids in the Great Lakes have different life history patterns, especially age of smolting (first leaving the river of their birth) and size and age of maturation (first returning to that river to spawn). How do different environments, like river and lake characteristics and abundance of predators and prey, lead to these different life histories? How do changing environments, like variations in temperature or in prey or predator abundance, affect life history traits? What is the underlying fitness function that leads to these life history patterns?

Age and size at maturity are important life history traits that have implications for individual fitness as well as for population growth rate. Several models have been developed to describe variation in age and size at maturity among or within taxa [1–11]. We compare our results with the results in these papers in more detail in the discussion section below.

Here we develop a model of optimal age and size at first reproduction, with the goal of developing a theory that uses accessible data, promotes understanding of diverse patterns, and is relevant to management decisions. Unlike other theoretical life history models, our approach uses individual-level formulations built from empirically based assumptions, rather than mathematical convenience.

We assume that age and size in general are related via a “growth function,” that fertility and size are related via a “fertility function,” and that survival and age are related via a “survival function.” We assume that life history traits emerge from the maximization of some fitness function. We examine how the choices of growth function, fertility function, survival function, and fitness function affect the predicted age and size at maturity.

In this context, environmental effects are usually brought into play via “reaction norms” [3]. A key thrust of this paper is to show a geometric method of studying reaction norms, and to use this method to see how reaction norms depend on the growth, survival, and fitness functions of the model. We show that different fitness functions lead to radically different reaction norms. We close by relating these geometric and analytical findings to actual Great Lakes salmonid data.

## Biology background and fitness functions

The basic building blocks of life history theory are *x*, *l*_*x*_, and *m*_*x*_, where *x* is the age in years since birth, *l*_*x*_ is the probability of surviving from birth to age *x*, and *m*_*x*_ is the average number of female offspring of a female of age *x*.

We focus on Great Lakes salmonids, especially Chinook Salmon. S1 Appendix presents the life history of the semelparous Chinook Salmon, along with a description of the parameter estimates we use in our simulations for the transitions from any life stage to the next.

We take birth (*x* = 0) as the day that eggs are laid and fertilized. This choice is different than the common choice of egg hatch to define the start of a generation; our choice facilitates analysis of the optimal timing of first reproduction in species where it can take many weeks for eggs to hatch. The survival probability *l*_*x*_ to age *x* is well-defined whether one uses continuous or discrete ages *x*. The age-specific fertility parameter *m*_*x*_ can be tricky for continuous ages *x*, but it is straightforward for salmon, which spawn at most once a year and on the same date (roughly October 1 for Great Lakes Chinook Salmon). In fact, using a growth function (size as a function of age) and a fertility function (number of eggs as a function of size), we will be able to work with continuous *x* in *m*_*x*_ when we need to.

Three classic fitness functions have been used to analyze life history characteristics: the intrinsic rate of natural increase *r*, the net reproductive rate *R*_0_, and the reproductive value of a female of age *x*, *V*_*x*_.

For discrete ages *x*, one can build a “life table” with columns for *x*, *l*_*x*_, and *m*_*x*_. One can also construct a system of linear difference equations from this table that keeps track of the size of each age group *x* over time. S2 Appendix presents such a system and its corresponding (Leslie) matrix representation. The bottom line of this approach is that *eventually* the size of each age group grows by the same factor λ each year, and therefore so does the total population. This multiplier λ is the annual population growth multiplier. The equation for λ in terms of the *l*_*x*_ and *m*_*x*_ is called the Euler Formula:

$$\frac{m_{1}l_{1}}{\lambda^{1}}+\frac{m_{2}l_{2}}{\lambda^{2}}+\cdots +\frac{m_{T}l_{T}}{\lambda^{T}}=1$$ (0.1)

where *T* is the maximum age attained. In mathematical terms, λ is the dominant eigenvalue of the Leslie matrix, and (0.1) is the (characteristic) equation that determines λ.

We usually replace the annual growth multiplier λ by its instantaneous counterpart *r*, where e^*r*^ *= λ* or *r =* ln λ. In this case, the Euler Formula (0.1) becomes:

$$\frac{m_{1}l_{1}}{e^{r}}+\frac{m_{2}l_{2}}{e^{2r}}+\cdots +\frac{m_{T}l_{T}}{e^{Tr}}=1.$$ (0.2)

Ecologists call the *r* computed from (0.2) the *intrinsic* or *instantaneous growth rate* of the population or the *intrinsic rate of natural increase*. It is one of the principal life history parameters of a population. It is also a principal candidate for a fitness function of a population, given its measure of how a population is growing over time. Note that *r* < 0 (or λ < 1) indicates a population shrinking in size over time, while *r* > 0 (or λ > 1) indicates an increasing population size.

A second candidate for the underlying fitness of a population is the net reproductive rate of the population, *R*_0_, sometimes called expected lifetime fertility [12]. When applied to individual fitness rather than population fitness, some use the term lifetime reproductive success [13]. This is the expected number of (female) offspring a newborn female will have in the course of her life. It measures the mean number of female offspring by which a female newborn will be replaced by the end of her life, and thus the factor by which a population increases from one generation to the next [14, p. 126]. Analytically, it is the probability *l*_*x*_ of surviving to age *x* times the expected number *m*_*x*_ of female offspring at age *x*, summed over ages *x*:

$$R_{0}=l_{1}m_{1}+l_{2}m_{2}+\cdots +l_{T}m_{T}$$ (0.3)

Comparing this expression with Euler’s Formula, we find: if λ = 1, *R*_0_ = 1. If λ > 1, then *R*_0_ > 1; and if λ < 1, *R*_0_ < 1. So, fitness measures *r* = ln *λ* and *R*_0_ are related. The parameter *R*_0_ is often treated as the population multiplier from one *generation* to the next [14, p. 128] and called the *net reproductive ratio* [15] or the *basic reproduction number*.

A third, more individual measure of fitness was introduced by R. A. Fisher [16] in answer to the question: what is the “present value” of the current and future offspring of a female of any age x? Fisher proposed the reproductive value of a female of age x:

$$V_{x}=\frac{1}{l_{x}}\left(l_{x}m_{x}+l_{x+1}m_{x+1}e^{-r}+l_{x+2}m_{x+2}e^{-2r}+\cdots +l_{T}m_{T}e^{-Tr}\right)$$ (0.4)

Fisher discounted the offspring in later years by the population growth *r* in those years, arguing that the impact of one’s offspring on a population decreases as the population size increases. In the formula for *V*_x_, each *l*_x+j_/*l*_x_ is simply the probability of surviving from current age *x* to age *x+j*.

Notice that *V*_0_ = 1 by Euler’s Formula and the definition that *l*_0_ = 1.

## Comparing and contrasting fitness functions

Given the standard assumption that organisms’ life history traits arise from maximizing some fitness function, we are interested in the timing of the first reproduction or “maturity”, specifically the age α and size *L*_α_ at maturity. We continue using Great Lakes salmonids as our focus group of species, in which case α is the age (in years since spawning and fertilization) at which a female first spawns. We compare and contrast the three fitness functions we have just defined: instantaneous per capita rate of population growth (*r*), net reproductive rate (*R*_0_), and reproductive value (*V*_x_) at any age *x*.

In this paper we focus on the semelparous case, where the fitness functions are especially simple. Recall that semelparous species, like Chinook and Coho Salmon (*O*. *kisutch*), die right after their first reproduction. Only one *m*_*x*_ is non-zero, the age of maturity *x*, which we denote by *α*. Thus, the three fitness functions for a *semelparous* species are simplified as:

$$\mathrm{Intrinsic}\;\mathrm{population}\;\mathrm{growth}\;\mathrm{rate}\;r:\;r=\frac{\ln(l_{\alpha }m_{\alpha })}{\alpha }$$ (0.5)

$$\mathrm{Net}\;\mathrm{reproductive}\;\mathrm{rate}\;R_{0}:\;R_{0}=l_{\alpha }m_{\alpha }$$ (0.6)

$$\mathrm{Reproductive}\;\mathrm{value}\;V_{x}\;\mathrm{for}\;x\leq \alpha :\;V_{x}=\frac{l_{\alpha }m_{\alpha }e^{-r\cdot }{}^{\left(\alpha -x\right)}}{l_{x}}.$$ (0.7)

We modify the notation to emphasize that these three fitness functions are functions of *α*: *r*(*α*), *R*_0_(*α*), and *V*_x_(*α*). Combining (0.5) and (0.7) yields:

$$V_{x}(\alpha )=\frac{e^{r(\alpha )x}}{l_{x}}$$ (0.8)

From (0.8) it is clear that any maturation age *α* that maximizes *r*(*α*) also maximizes *V*_x_(*α*). These two fitness functions give the same optimal age at maturity, as we show I n S3 Appendix for the more general case [17,18]. From now on we will only work with *r*(*α*) and *R*_0_(*α*).

We first compare *r*(*α*) and *R*_0_(*α*). Combining (0.5) and (0.6) yields:

$$r(\alpha )\alpha =\ln R_{0}(\alpha ).$$

Assuming, for a moment, differentiability of *r*(*α*) and *R*_0_(*α*),

$$r(\alpha )+r^{\prime }(\alpha )\alpha =\frac{R_{0}{}^{\prime }(\alpha )}{R_{0}(\alpha )}$$

At the maximizer of *r*(*α*), *r*’(*α*) = 0 and so:

$$\mathrm{When}\;r^{\prime }(\alpha )=0,\;r(\alpha )=\frac{R_{0}{}^{\prime }(\alpha )}{R_{0}(\alpha )}.$$ (0.9)

If *r*(*α*) > 0, then *R*_0_*’*(*α*) > 0 and *R*_0_(*α*) is increasing; the maximizer of *R*_0_(*α*) is greater than the maximizer of *r*(*α*) when the population growth rate *r*(*α*) is positive. In other words, the age of maturation that maximizes *R*_0_ will be *greater* than the age of maturity that maximizes *r* when *r* > 0. Similarly, when population size is decreasing (*r* < 0), the age of maturation that maximizes *R*_0_ will be *less* than the age of maturity that maximizes *r*. This is a general result that follows from the definitions of *r* and *R*_0_ for semelparous populations and does not really require the differentiability assumption. We will show a graphical version of this result below.

## Finding optimal age and size at first reproduction

We take an analytical approach to deriving the optimal size and age at maturation, using *r* and *R*_0_ as the underlying fitness functions. We then display the results geometrically. We use survival and fertility functions to write our objective functions *r* and *R*_0_ as functions of size and age at maturity and then draw their level sets (or isopleths) in age-size space, curves on which the fitness function takes on the same value, like the iso-temperature curves on a weather map or the indifference curves in consumer theory. One draws these iso-fitness curves in age-size space along with the growth curve for the population under study. To find the point of *highest* fitness on the growth curve, one looks for the highest fitness level curve that touches the growth curve. At this optimal fitness point, the iso-fitness curve will, in general, be tangent to the growth curve. This process is analogous to the way microeconomics students find the level set (“indifference curve”) of highest utility that intersects a given budget line.

This geometric approach allows us to see how varying background ecological parameters, like food supply and predation, that alter the population growth curve lead to different optimal points of age/size at maturity and thus generate the classic reaction norms.

### Survival as a function of age

We assume that age-specific survivorship *l*_*x*_ is a product of three factors: the probability *l*_*y*_ of surviving the early life history period in the stream (from fertilization to reaching the lake as a juvenile (smolt) at age *y*), the probability of survival from entering the lake as a smolt at age *y* to first reproduction at age *α*, and the probability *q* of surviving the journey upstream to the egg-laying site. For simplicity we make the standard assumption that annual survival in the lake from smolt age *y* to maturation age *α* is a constant *s*. We express annual survival probability *s* as an *instantaneous mortality rate z*: *s = e*^*−z*^, or *z* = −ln *s*. Therefore, the probability at fertilization of surviving to first reproduction at age *α* is:

$$l_{\alpha }=l_{y}e^{-z(\alpha -y)}q$$ (0.10)

In the numerical computations behind our figures, we use *l*_*y*_ = 0.00953, *s* = 0.70, so that *z* = 0.357 yr^-1^, *y* = 0.701 yr, and *q* = 0.667. See S1 Appendix for details.

### Fertility as a function of size

The number of eggs per female increases with female body size in a wide variety of taxa, including fish [1, p. 126]. We assume that fertility at age *x* is a power function of female length *L*_*x*_ at age *x*:

$$m_{x}=A{\left(L_{x}\right)}^{b}$$ (0.11)

for some constants *A* and *b*. This assumption is well supported for fish [19,20]. The parameter *A* includes the assumption that half the eggs produced are female. The parameter *b* includes the fact that weight and volume are related.

In the numerical computations behind our figures, we use *A* = 0.5*e^−4.190^ ≈ 0.00757 and *b* = 1.891. See S1 Appendix for details.

### *R*_0_ as a function of age and length at maturity

If we substitute the functions for survivorship (0.10) and fertility (0.11) into the definition (0.6) for *R*_0_, the result is *R*_0_ as a function of age α and size *L*_α_ at maturity:

$$R_{0}=[ql_{y}\;e^{zy}A]{\left(e^{-z}\right)}^{\alpha }{\left(L_{\alpha }\right)}^{b}$$ (0.12)

It is helpful to work with the level sets of ln(*R*_0_) in terms of age and log(length). Taking the natural logs of both sides of (0.12) and solving for ln *L*_*α*_ in terms of *α*, we find

$$\ln L_{\alpha }=\frac{1}{b}\left[\ln R_{0}–\ln\left(ql_{y}e^{zy}A\right)\right]+\alpha \frac{z}{b}$$ (0.13)

**Fig 1:**
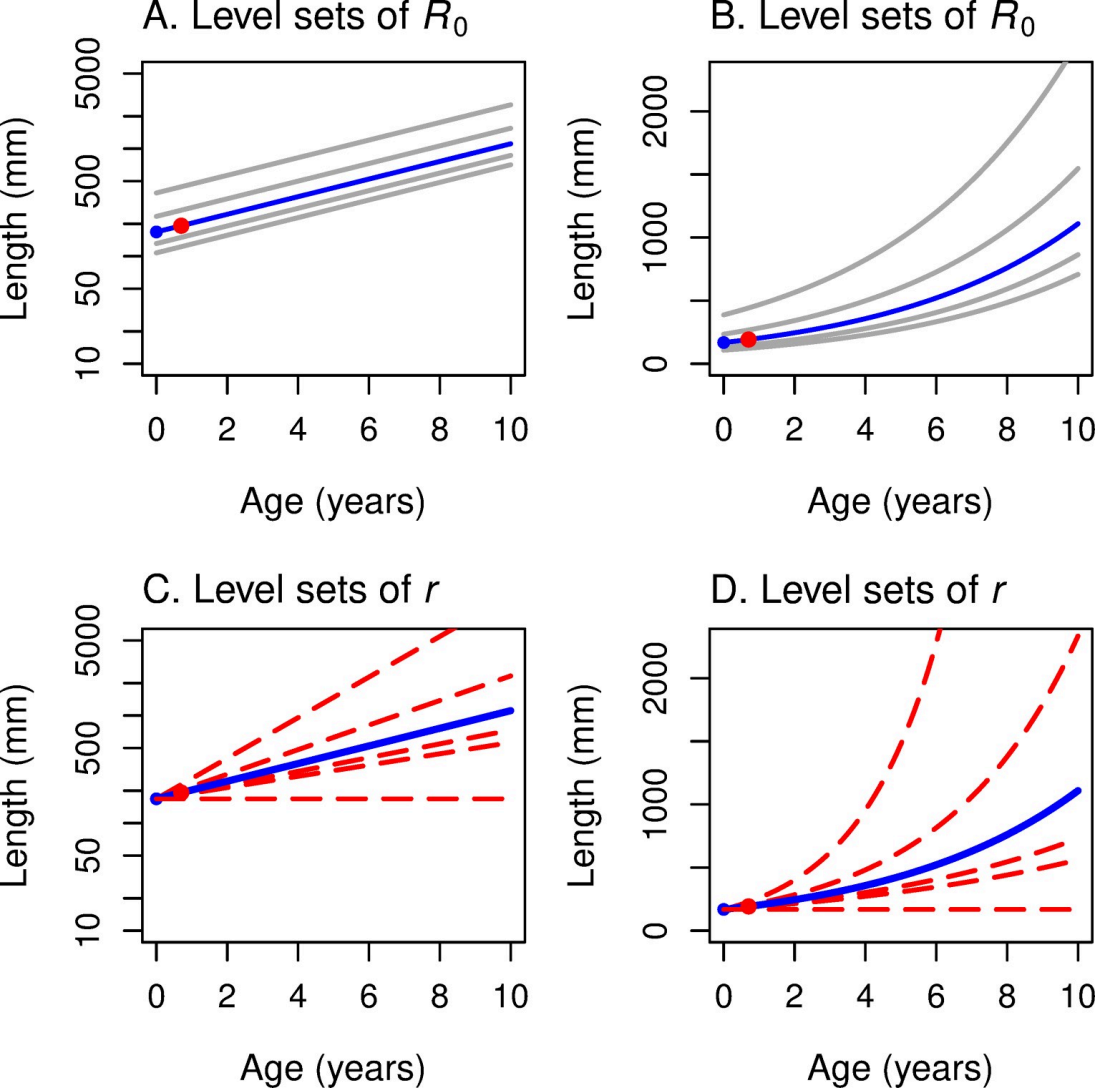
Level sets of fitness functions *R*_0_ and *r* are straight lines in age/log-length space. Level sets of fitness function *R*_0_ are parallel lines in maturation age/log-length space (A) and concave-up curves in maturation age/linear-length space (B). Level sets of fitness function *r* are linear rays emanating from a common point in maturation age/log-length space (C) and concave-up curves emanating from a common point in maturation age/linear-length space (D). That common point, colored blue in all four panels, is the y-intercept *L*_α0_ of the level set *R*_0_ = 1, or equivalently of level set *r* = 0 (the blue line). The red point in all four panels is the point on *R*_0_ = 1 or *r* = 0 that corresponds to maturation age α equal to smolt age, a point that plays a key role in our analysis.

As illustrated in Fig 1, the level sets of ln *R*_0_ in age-log(length) space are straight lines with a common slope *z*/*b* and with an intercept that depends on *R*_0_. When *R*_0_ = 1, the *α* = 0 intercept is:

$$\ln L_{\alpha }{}_{0}=-\frac{1}{b}\left[\ln\left(ql_{y}A\right)+zy\right]$$ (0.14)

We write *L*_*α*0_ for the *α* = 0 intercept of the *R*_0_ = 1 level set, an expression that plays a central role in our geometric analysis (even though maturation will never occur at *α* = 0):

$$L_{\alpha }{}_{0}={\left(ql_{y}e^{zy}A\right)}^{\frac{-1}{b}}$$ (0.15)

In age-length space, we observe that, by exponentiating both sides of (0.13), the level curves of *R*_0_ are positively-sloped exponential curves, as shown in Fig 1.

### *r* as a function of age and length at maturity

We next substitute the functions for survivorship (0.10) and fertility Eq (0.11) into the definition (0.5) for *r* to write *r* as a function of age α and size *L*_*α*_:

$$r=-z+\frac{b}{\alpha }\left[-\ln\left(L_{\alpha }{}_{0}\right)+\ln\left(L_{\alpha }\right)\right],$$ (0.16)

where ln(*L*_*α*0_) is given by (0.14). We can describe the level sets of *r* in age-log(length) space by solving (0.16) for ln(*L*_*α*_):

$$\ln\left(L_{\alpha }\right)=\alpha \left(\frac{r+z}{b}\right)+\ln\left(L_{\alpha }{}_{0}\right)$$ (0.17)

On each level set of *r*, ln(*L*_*α*_) is a linear function in α with slope (*r+z*)/*b* and intercept ln(*L*_*α*0_), as illustrated in Fig 1. These “concurrent” lines all emanate from their common horizontal intercept, ln(*L*_*α*0_). They are positively sloped as long as *r* > *−z*, that is, as long as the growth rate exceeds the death rate.

If we exponentiate both sides of (0.17), we find that for each value of *r*, the *L*_*α*_ versus *α* curves form a family of exponential curves (Fig 1). Note that these curves emanate from a common horizontal intercept, *L*_*α*0_.

$$L_{\alpha }=L_{\alpha }{}_{0}\;\exp\left(\frac{\left(r+z\right)\alpha }{b}\right)$$ (0.18)

The level curve for *r* = *-z* is a horizontal line at *L*_*α*_ = *L*_*α*0_; for *r* < *-z*, the level curves of *r* slope downward with age.

### Two reference points for level sets of *R*_0_ and *r*

The level sets of *r* and of *R*_0_ as a function of age *α* and length *L*_*α*_ at maturity play a key role in our construction and analysis of reaction norm curves in (*α*, *L*) space. We have just seen that–in (*α*, ln *L*) space–the level sets of *R*_0_ are parallel lines with common slope *z*/*b*, and the level sets of *r* are rays emanating from the point (0, ln *L*_α0_), where *L*_α0_ is given by (0.14).

For reference, we note that the line *R*_0_ = 1 coincides with line *r* = 0, with equation

$$\ln\left(L_{\alpha }\right)=\ln\left(L_{\alpha }{}_{0}\right)+\alpha \left(\frac{z}{b}\right).$$

This is a good reference line for our geometric analysis. In particular, this line emanates from (0, ln *L*_α0_). To find another reference point on this common line, let’s work with age *α* = *y*, the age of smolt (highly improbable biologically, but handy geometrically). On R_0_ = 1, 1 = *l*_*y*_ *m*_*y*_. So, the fertility at age *y* must be *m*_*y*_ = 1/*l*_*y*_. Using the fertility function (0.11) that relates length and fertility, we find that the length at age *y* that would result if *R*_0_ = 1 is given by:

$$\ln\left(L_{y}{}^{*}\right)=-\frac{1}{b}\ln\left(qAl_{y}\right)\;\mathrm{for}\;\mathrm{age}=y\;\mathrm{and}\;R_{0}=1,$$ (0.19)

so that this point, along with the slope *z/b* and age *y*, determines the age-0 intercept ln *L*_α0_:

$$\ln\left(L_{\alpha }{}_{0}\right)=\ln\left(L_{y}{}^{*}\right)–y\left(\frac{z}{b}\right)$$ (0.20)

Level sets for *R*_0_ = 1 (and *r* = 0) must go through the point (*y*, *L*_y_*) in age-length space. This reference point is indicated in red in our figures.

### Effects of changing mortality rate *z*

In our geometric analyses, we want to understand how the level sets of *r* and *R*_0_ change as the instantaneous mortality rate *z* changes. For the parallel level sets of *R*_0_ with slope *z*/*b*, as *z* increases, so does the slope of all the level lines. For the concurrent level sets of *r*, their common intercept ln(*L*_*α*0_) decreases as *z* increases. Within each concurrent “spray” of lines, as *z* increases, the slope (*r+z*)/*b* of an *r*-level line increases. Alternatively, as *z* increases, a line with a given slope will have a lower *r-*value.

## Optimal age and size of maturation using growth functions

### General formula for optimal age and size at maturation

Our goal has been to compute optimal age and size of maturation for semelparous species given various choices of fertility, survival, growth and fitness functions. We ask for the age and size that maximize a given fitness function subject to an underlying growth relationship. In the last section we began to describe the geometry underlying this process; in this section we carry out the analysis. The straightforward way of accomplishing this is to substitute the selected growth function *L* = *L*(*α*) into the fitness function and then maximize the resulting function of the one variable *α*. (Alternately, one can use a Lagrange multiplier approach [21].)

For maximizing net reproductive rate *R*_0_, use (0.12) to write *R*_0_(α,*L*(α)) as a function only of *α*, take the derivative with respect to *α*, set it equal to 0 and solve. We show in S4 Appendix that one finds the relationship:

$$\frac{L^{\prime }(\alpha )}{L(\alpha )}=\frac{d(\ln L(\alpha ))}{d\alpha }=\frac{z}{b}$$ (0.21)

This shows that the age and length that maximize *R*_0_ occurs at the point (α*, ln L*) in age-log length space where the growth trajectory has slope *z*/*b*. This represents the highest *R*_0_ level line that meets any given growth trajectory. This general formula (0.21) can be used with any growth function *L*(*α*) to determine the optimal age and length at maturation that maximizes *R*_0_.

Multiply Eq (0.21) through by the fertility parameter *b*. Then, (0.21) implies that the optimal size and age of maturity occur where the marginal fertility from a little more growth balances with the marginal mortality due to the delay incurred by that additional growth.

For maximizing population growth rate *r*, do the same with (0.16). We show in S5 Appendix that one finds the relationship:

$$\frac{L^{\prime }(\alpha )}{L(\alpha )}=\frac{d\left(\ln L(\alpha )\right)}{d\alpha }=\frac{\ln L-\ln L_{\alpha 0}}{\alpha }$$ (0.22)

This shows that the age and length that maximize *r* occurs at the point (α*, ln L*) in age-log length space where the growth trajectory has slope (ln *L*–ln *L*_α0_)/*α*. This represents the highest *r* level line that originates as a ray from the point (0, ln *L*_α0_) and meets any given growth trajectory. This general formula (0.22) can be used with any growth function *L*(*α*) to determine the optimal length at maturation that maximizes *r*.

Next, we examine two specific functions for growth in the lake (length as a function of age): linear growth in length and nonlinear asymptotic growth in length. Fish growth is indeterminate. The most commonly used growth function is the von Bertalanffy growth curve [22], where length increases as age increases but at a decreasing rate and eventually levels off to a limiting size.

### Linear growth

We start by working with the simpler linear growth curves, which have a constant rate of increase in length. Some fish species have approximately linear growth in length as juveniles [4,23–28]:

$$L_{x}=c+k\;x,$$ (0.23)

where *L*_*x*_ is length [mm] at age *x* [years], *c* is the length [mm] at age 0, and *k* is the growth rate [mm/year].

#### *R*_0_ with linear growth

To study semelparous species with linear growth and fitness function *R*_0_, we combine (0.21) and (0.23) to find:

$$L=\frac{b}{z}k$$ (0.24)

$$\alpha =\frac{b}{z}-\frac{c}{k}$$ (0.25)

The optimal length at maturity, *bk*/*z*, is proportional to the linear growth rate *k* and to the exponent *b* relating length to fecundity, and inversely proportional to the instantaneous mortality rate *z*, and—surprisingly—is independent of initial length *c*. For the special case of *c* = 0 in the linear growth function, the optimal age α of maturity is simply *b*/*z*, independent of the growth rate *k*. It is not surprising that optimal age of maturity is inversely proportional to the mortality rate *z*. It is a general tenet of evolutionary biology that the more dangerous life is, that is, the higher the probability of an earlier death, the more it makes sense to speed up the timetable of reproduction.

**Fig 2:**
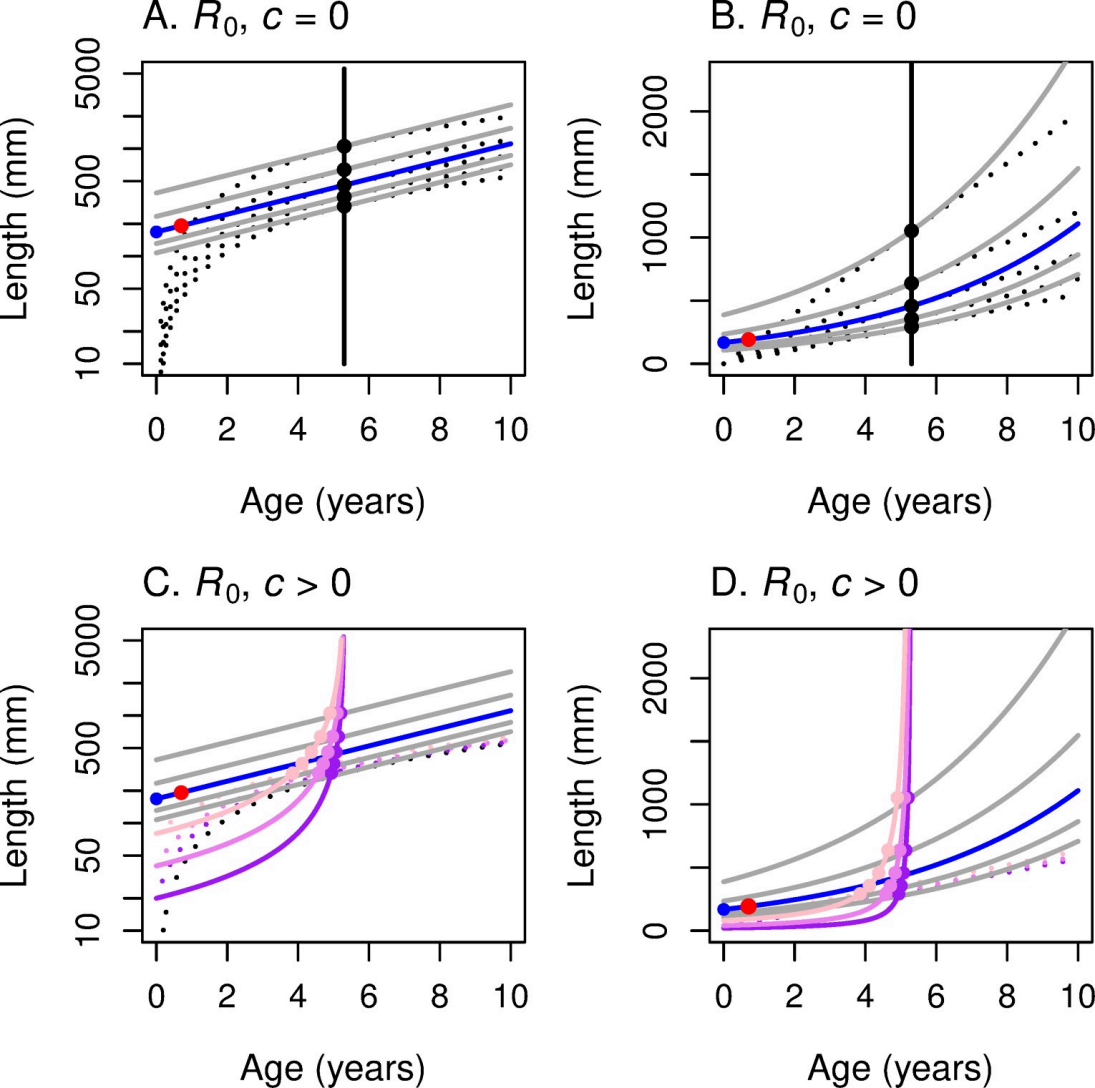
These panels illustrate the construction of the maturation reaction norm MRN(*R*_0_) for linear growth. For linear growth: *L* = *c* + *k x*, where *L* is length, *x* is age in years, *k* is the growth rate, and *c* the size at age 0. The underlying fitness function is *R*_0_ for all four panels. (A, C) The *y*-axis represents log-length so that the iso-fitness curves (solid grey lines) are straight lines. (B, D) The *y*-axis represents linear length so that the linear growth curves (black dotted lines) are straight lines. Size *c* at *x* = 0 is set to 0 in the first row; *c* > 0 in the second row (purple: 20 mm; violet: 40 mm; pink: 80 mm). (The analysis is much simpler for *c* = 0.) Higher growth curves correspond to higher values of the growth rate *k*. For each growth curve, one finds the highest iso-fitness curve that intersects it. The locus of such points as *k* varies traces out the reaction norm (solid black curve for *c* = 0, colored lines for *c* > 0). The reaction norm is mostly vertical when *R*_0_ is the fitness function (versus perfectly horizontal when *r* is the fitness function; Fig 3). Fig 4 summarizes and consolidates this information.

At least as important as computing the optimal *α* and *L*_*α*_ for any growth and mortality rate is ascertaining how these optimal values change as the underlying environment changes. In Fig 2 we put our fitness graphs and growth graphs together to see how the optimal age and size at maturation depend on the parameters of the problem.

Fig 2A shows the parallel linear level sets of *R*_0_ as function of age and log-length, as in Fig 1A. We superimpose on these the graphs of linear growth functions (0.23) for different values of *k* for *c* = 0. These growth curves–linear in age/length space–are non-linear logarithmic curves in age/log-length space. For any particular growth function (dotted line), the solid dot indicates the age/log-length point where the growth line meets the highest level set of *R*_0_ and is therefore tangent to it. We see in Fig 2A that for different values of *k*, that is, for different linear growth rates, the optimal age α of maturity is constant–as we saw analytically in the last paragraph, independent of the slope *k* of the growth curve. Think of *k* as depending on something like food availability that has an impact on growth. For a semelparous fish with linear growth in length and fitness function *R*_0_, optimal age of maturity does not change as food availability changes; but the optimal length at maturity changes dramatically.

These curves that trace out the optimal values of age and length (or log-length) at maturity as an environmental parameter changes are called “reaction norms,” in our case, “maturation reaction norms” (MRNs). We denote the maturation reaction norms that maximize *R*_0_ or *r* as MRN(*R*_0_) and MRN(*r*), respectively. A key thrust of this paper is to show a geometric method of studying reaction norms, and to use this method to see how reaction norms depend on underlying components of the model.

Fig 2B shows the corresponding result in age vs. length, as we did in Fig 2A; in this plot the level curves of *R*_0_ are exponential curves and the growth curves are rays from the origin.

What do the corresponding maturation reaction norms look like for *c* > 0? Geometrically, we are shifting up the array of growth curves emanating from the origin in Fig 2A and 2B to an array of growth curves emanating from (0, *c*) as in Fig 2C and 2D. Analytically, for a fixed *c* > 0, we think of the Eqs (0.24) and (0.25) as defining a curve parameterized by *k*. To get the equation of this MRN(*R*_0_) curve, eliminate *k* from these equations:

$$L_{c}{}^{*}\left(\alpha \right)=\frac{bc}{b-\alpha z}\;\mathrm{for}\;0\leq \alpha \leq \frac{b}{z}$$ (0.26)

We write *L* = L*(*α*) to describe the MRN curve, reserving the unstarred *L* = *L*(*α*) for the underlying growth curve. We note that L*_c_(0) = *c*, that L*_c_*’*(0) = *zc*/*b*, and that L*_c_(*α*) has a vertical asymptote at *α* = *b*/*z* and is increasing from *α* = 0 to *α* = *b*/*z*. Reaction norm curves for three positive values of *c* are drawn in Fig 2C and 2D. Note that these curves are still mostly vertical since they are asymptotic to the line *α* = *b*/*z*, the MRN(*R*_0_) curve for the *c* = 0 case.

Compare Fig 2D (*c* > 0, *R*_0_) with Fig 2 in Perrin and Rubin [4], derived from simulations in which the authors are treating the iteroparous case and hold *b* = 3.

#### *r* with linear growth

For maximizing population growth rate *r* subject to linear growth, we combine the equation defining optimal length (0.22) and the linear growth function (0.23) to find:

$$\ln L_{\alpha }=\frac{\alpha k}{c+\alpha k}+\ln L_{\alpha 0}$$ (0.27)

For the special case *c* = 0, in which the growth curves are rays from the origin with slope *k*, the expression (0.27) is simplified:

$$L_{\alpha }=e\cdot L_{\alpha 0}\;\mathrm{for}\;c=0$$ (0.28)

The optimal length at maturity to maximize *r* is independent of *α* and independent of growth rate *k*. The MRN(*r*) is a horizontal line in age-length space, in contrast to the vertical line for MRN(*R*_0_) when *R*_0_ is the fitness measure.

When *c* > 0, eliminating *k* in expression (0.27) becomes:

$$\ln(L_{\alpha })=1–\frac{c}{L_{\alpha }}+\ln(L_{\alpha 0})\;\mathrm{for}\;c>0$$ (0.29)

where *L*_α0_ is given by (0.15). Note that expression (0.29) is also independent of *α*. Knowing the optimal length at maturity *L*_α_, the corresponding age at maturity *α* is found using the linear growth rate (0.23):

$$\alpha =\frac{L_{\alpha }-c}{k}$$ (0.30)

**Fig 3:**
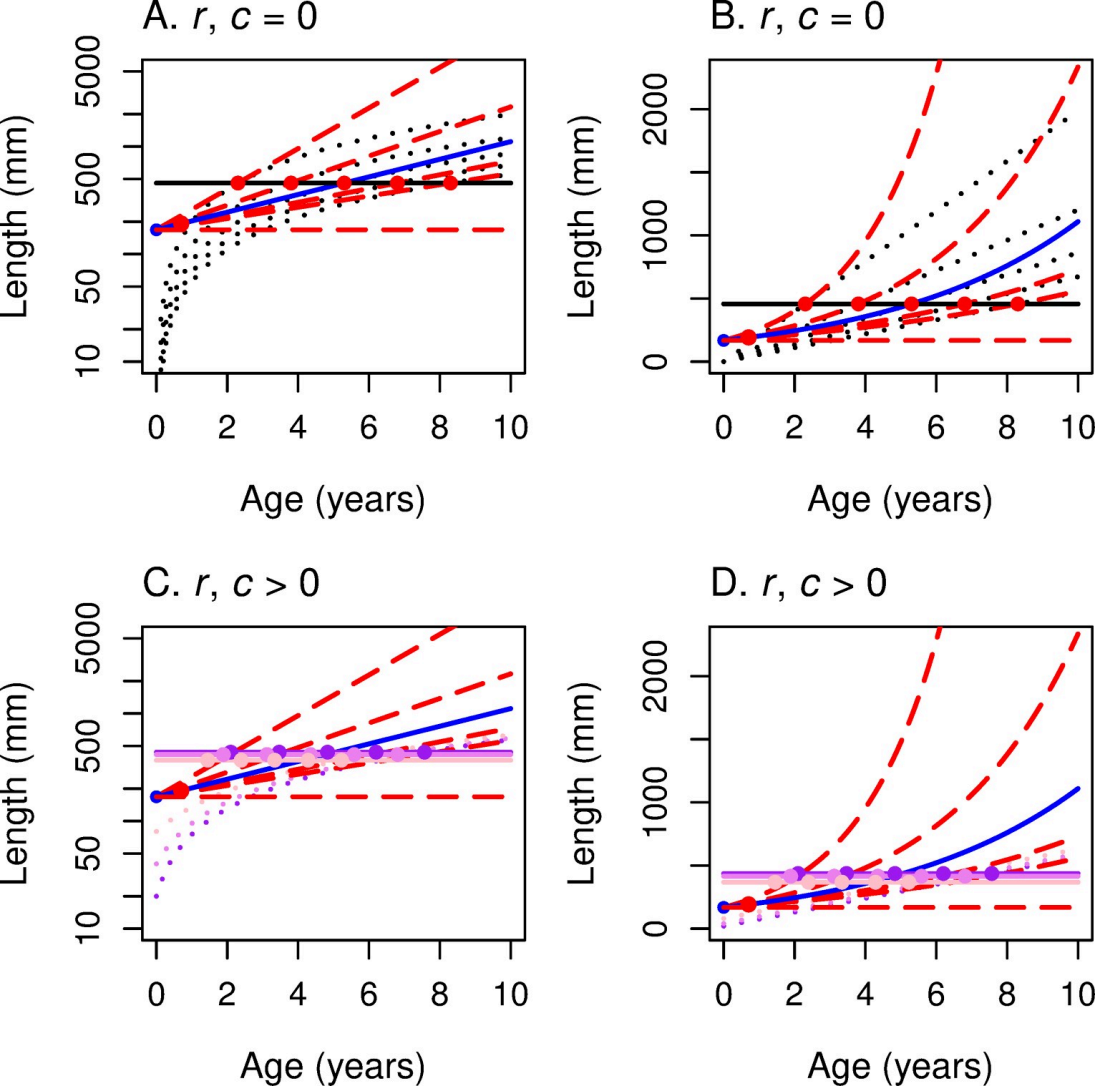
These panels illustrate the construction of the maturation reaction norm MRN(*r*) for linear growth. For linear growth: *L* = *c* + *k x*, where *L* is length, *x* is age in years, *k* is the growth rate, and *c* the size at age 0. *r* is the underlying fitness function for all four panels. (A, C) The *y*-axis represents log-length so that the iso-fitness curves (red-dashed lines) are straight lines. (B, D) The *y*-axis represents linear length so that the growth curves (black dotted lines) are straight lines. Size *c* at *x* = 0 is set to 0 in the first row; *c* > 0 in the second row (purple: 20 mm; violet: 40 mm; pink: 80 mm). Higher growth curves correspond to higher values of the growth rate *k*. For each growth curve, one finds the highest iso-fitness curve that intersects it. The locus of such points as *k* varies traces out the reaction norm (solid black line for *c* = 0, colored horizontal lines for *c* > 0). The reaction norm is perfectly horizontal when *r* is the fitness function (versus mostly vertical when *R*_0_ is the fitness function; Fig 2). Fig 4 summarizes and consolidates this information.

The geometry behind these calculations for fitness measure *r* and linear growth (0.23) is shown for various levels of *c* in age-log length space (Fig 3A and 3C) and in age-length space (Fig 3B and 3D). For *c* = 0, the graphs of the growth curves for different values of the growth constant *k* are rays from the origin, designated by the dotted black lines in Fig 3A. The level sets of *r* in age-length space (Fig 3B) are the exponential curves (the long-dash curves) emanating from the same point on the horizontal axis, as we saw in Fig 1D. The constrained maxima occur at the tangencies, all of which lie on the MRN(*r*), the solid horizontal line in Fig 3A and 3B. As growth rate *k* increases, for example via increased food supply, optimal age of maturation decreases but the optimal length at maturation remains constant. The MRN(*r*) lines remain straight lines even for *c* > 0 (Fig 3C and 3D). In contrast, recall that the MRN(*R*_0_) lines become curved for *c* > 0 (Fig 2C and 2D).

Note that, for fitness function *r*, mortality *z* enters the expressions for optimal size (0.27) and age (0.30) only in the ln(*L*_α0_) term. Furthermore, *z* appears in the expression (0.15) for ln(*L*_α0_) only in the product *zy*. So, if one assumes *y* = 0, then *z* does not enter at all. Some authors [1,29] work with *y* = 0 as a simplifying assumption for the hypothesis that early mortality occurs in a small time interval close to age 0. Our analysis shows that such an assumption leads to the (perhaps counter-intuitive) conclusion that optimal size and age at maturity are independent of the juvenile mortality parameter z, but they are dependent on the proportion (*p* = *l*_*y*_) surviving the early mortality period.

#### Comparing maturation reaction norms for *R*_0_ and *r* for linear growth

**Fig 4:**
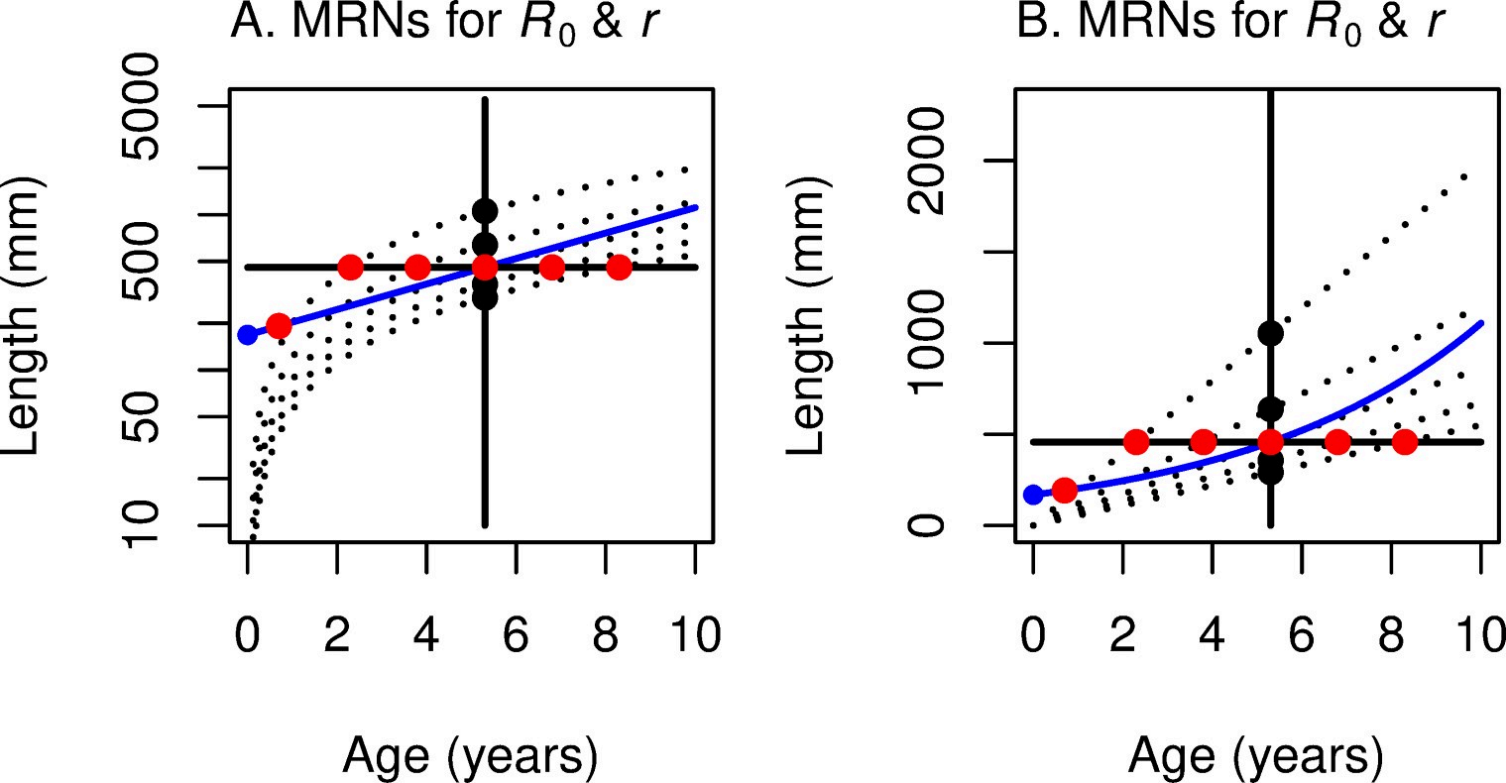
Growth trajectories with maturation reaction norms for maximizing *r* (horizontal line) and *R*_0_ (vertical line). Growth trajectories (dotted lines) show the linear growth function *L* = *c* + *k x*, with *c* = 0; red dots show where *r* is maximized; black dots show where *R*_0_ is maximized along the growth trajectory. (A) With log-length on the vertical axis, the growth trajectories are curved, and the underlying iso-fitness lines are straight lines (shown only as the blue line corresponding to *R*_0_ = 1 or *r* = 0; see Fig 2A and Fig 3A). (B) With linear-length on the vertical axis, the growth trajectories are straight lines, and the underlying iso-fitness lines are curved lines (blue line corresponds to *R*_0_ = 1 or *r* = 0; see Fig 2B and Fig 3B). In both panels, the MRN for fitness function *r* is horizontal (optimal maturation *size* does not change as growth rate *k* varies), while the MRN for fitness function *R*_0_ is vertical (optimal maturation *age* does not change as growth rate *k* varies).

Fig 4 shows both MRN(*R*_0_) and MRN(*r*) on the same age/log-length graph and age/length graph for the case of semelparous species and linear growth in length (with *c* = 0). The solid, black, horizontal line is MRN(*r*); the solid, black, vertical line is MRN(*R*_0_). The short-dash black lines arising from the origin represent different growth trajectories. Following along a single growth trajectory, one can see that the maturation age that maximizes *r* (red point) is less than the maturation age that maximizes *R*_0_ (black point) when *r* > 0 and *R*_0_ > 1 (above and to the left of the blue line). But when *r* < 0 and *R*_0_ < 1 (below and to the right of the blue line), this reverses, and the maturation age that maximizes *R*_0_ is less than the maturation age that maximizes *r*. The maturation ages are identical when *r* = 0 and *R*_0_ = 1 (on the blue line). This is a geometric example of the analytical result concluding section 3.

### von Bertalanffy growth

The most common growth function used in models of fish life history is the von Bertalanffy growth curve, which assumes that early growth is rapid but tapers off to an asymptotic size *L*_*∞*_ [30]. Although von Bertalanffy growth has been criticized by some researchers [25,26,27,31], it has become a standard for describing annual growth of fishes [3,22,32,33]. Beyond fish, Kooijman [34] fitted the von Bertalanffy equation to 250 species in nine phyla.

We will work with a post-smolt von Bertalanffy curve, with growth rate *k* in the lake until spawning and death.

$$L_{x}=L_{\infty }\left(1-Ce^{-k\left(x-y\right)}\right)\;\mathrm{for}\;\mathrm{ages}\;x\leq \mathrm{smolt}\;\mathrm{age}\;y.$$ (0.31)

Here *C* = 1 –*L*_*y*_/*L*_∞_, where *L*_*y*_ is the length at smolting, when the female enters the lake at age *y*, and *k* is the von Bertalanffy growth coefficient [units: year^-1^], analogous to the *k* [units: mm/yr] in the linear growth curve (0.23). A semelparous salmon that matures at age *α* after (*α* - *y*) years in the lake, will have length

$$L_{\alpha }=L_{\infty }\left(1-Ce^{-k\left(\alpha -y\right)}\right)$$ (0.32)

In the numerical calculations behind our figures, we take *y* = 0.701 yr, *L*_*y*_ = 80 mm, *L*_*∞*_ = 1019 mm, and so *C* = 0.921 (see S1 Appendix for details).

#### *R*_*0*_ with von Bertalanffy growth

To find the optimal *α* and *L* for semelparous species with von Bertalanffy growth using fitness function *R*_0_, we combine the *R*_0_ optimality Eq (0.21) and the von Bertalanffy Eq (0.32). The results (S6 Appendix) are values that depend on growth rate *k*.

$$\begin{array}{c} \alpha =y+\frac{1}{k}\left[\ln\left(C\left(\frac{bk+z}{z}\right)\right)\right]\\ L_{\alpha }=L_{\infty }\left(1-\frac{z}{bk+z}\right)=L_{\infty }\left(\frac{bk}{bk+z}\right) \end{array}$$ (0.33)

For the special case where *C* = 1, *b* = 3, and *y* = 0, the top equation in (0.33) is identical to the equation derived by Roff [1], his equation (4.81), and by Morita et al. [11] for maximizing lifetime fertility *R*_0_.

Rewrite the second equation in (0.33) as:

$$\frac{L_{\alpha }}{L_{\infty }}=\frac{1}{1+\frac{z}{bk}}.$$ (0.34)

Eq (0.34) gives a relationship between the two Beverton-Holt invariants: (*z*/*k)* and (L_α_/L_∞_). See [35] and our discussion section below. Eq (0.34) suggests that the exponent *b* in the fertility function (0.11) should be included among the life-history invariants; more specifically, (*z*/*bk*) should replace (*z*/*k*). Including the parameter *b* preserves the invariant as a dimensionless number, while accounting for differences among taxa in the size-dependence of the allocation to reproduction.

Before we examine the reaction norm, we ask: How do the optimal α* and L* vary with *k* in (α,L) space? We write L*_α_(*k*) and α*(*k*) for the *R*_0_-maximizing expressions in (0.33). Since

$$\frac{dL{*}_{\alpha }}{dk}=L_{\infty }\frac{bz}{{\left(bk+z\right)}^{2}}>0,$$

**Fig 5:**
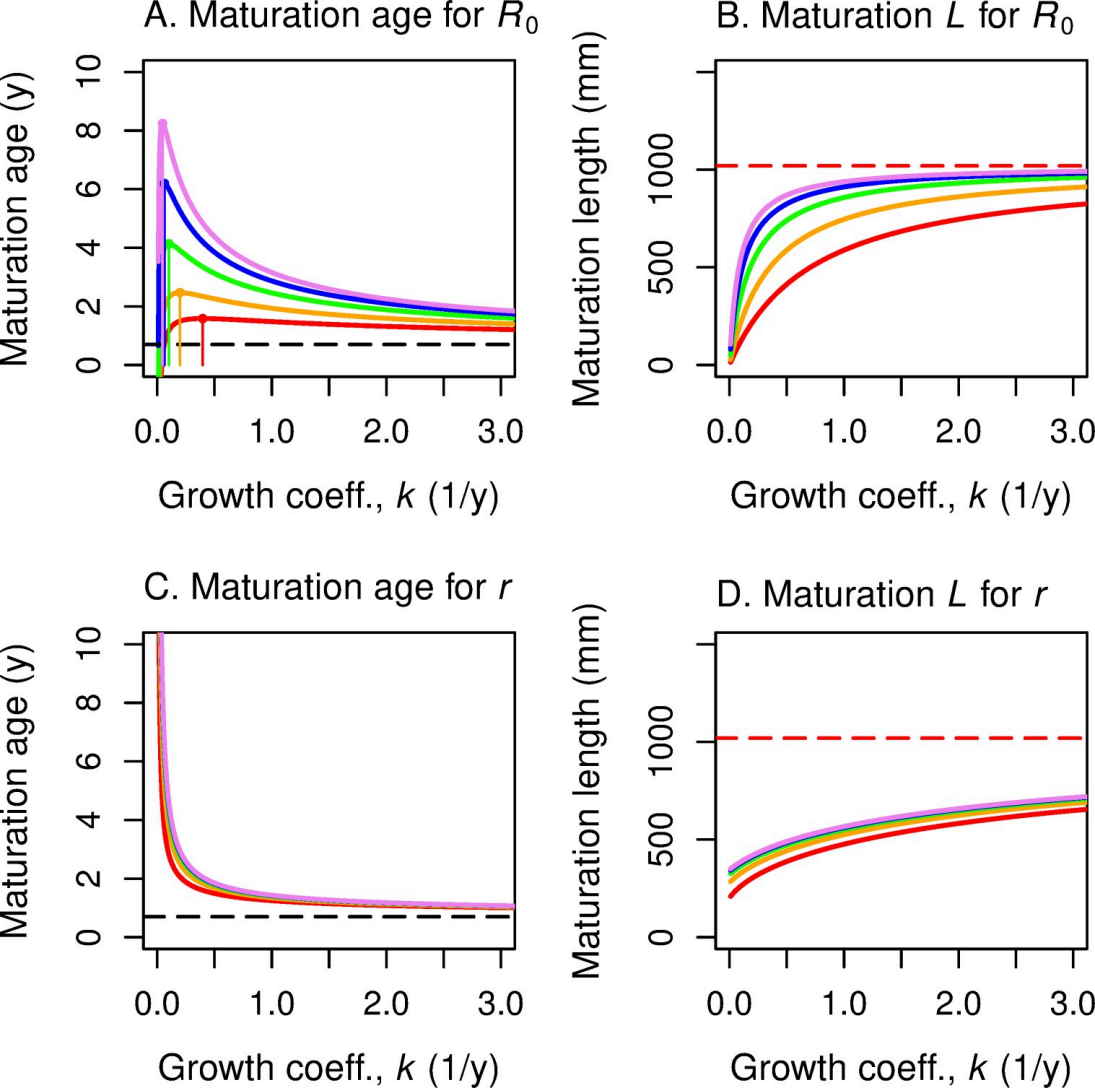
Optimal maturation age and length versus von Bertalanffy growth coefficient, for five survival rates. In all four panels, the horizontal axis corresponds to the growth rate *k* in the non-linear von Bertalanffy growth curve. The vertical axis presents the optimal maturation *age* on the left two panels (A, C) and the optimal maturation *length* on the right two panels (B, D), for each value of *k*. For the top two panels the underlying fitness function is *R*_0_; for the bottom two panels the underlying fitness function is *r*. In each panel, optimal age and size curves are drawn for five values of survival rates *s* (red: 0.25; orange: 0.50; green: 0.70; blue: 0.80; violet: 0.85). As the annual survival rate increases from *s* = 0.25 to 0.85, the optimal maturation age and length increase for a given *k*. In panels A & C, the horizontal black dashed line indicates the age at smolting; the colored points at the peak of the vertical lines indicate the maximum value of optimal maturation ages for a given annual survival *s*. In panels B and D, the horizontal red dashed line represents the asymptotic length of the von Bertalanffy growth function.

L*_α_ is a monotone increasing function of *k*; in fact, L*_α_ converges toward *L*_*∞*_ as *k* increases. See Fig 5B. However,

$$\frac{d\alpha *}{dk}=\frac{\frac{\kappa -1}{\kappa }-\ln C-\ln\kappa }{k^{2}},\;\mathrm{where}\;\kappa \equiv \frac{bk+z}{z}\geq 1.$$

The numerator in this expression starts positive at–ln *C* (since 0 < *C <* 1), increases monotonically (since its derivative is positive) and is eventually negative (since–ln κ → −∞). Therefore, α*(*k*) has an inverted U-shaped graph. It increases to a peak and then decreases as *k* grows. Fig 5A and 5B presents graphs of these parameter curves for reasonable values of the parameters.

The inverted U-shaped graph of α*(*k*) makes intuitive sense (Fig 5A). In a resource constricted environment, say when food is scarce (low *k*), the ability to survive and produce more eggs is limited. In this regime, one should respond to still lower resource availability by reproducing earlier (“while one still can”). On the other hand, if food is plentiful and getting more so and one is near maximum size, it makes no sense to delay reproduction for the benefit of a little extra body weight and a tiny increase in fecundity.

**Fig 6:**
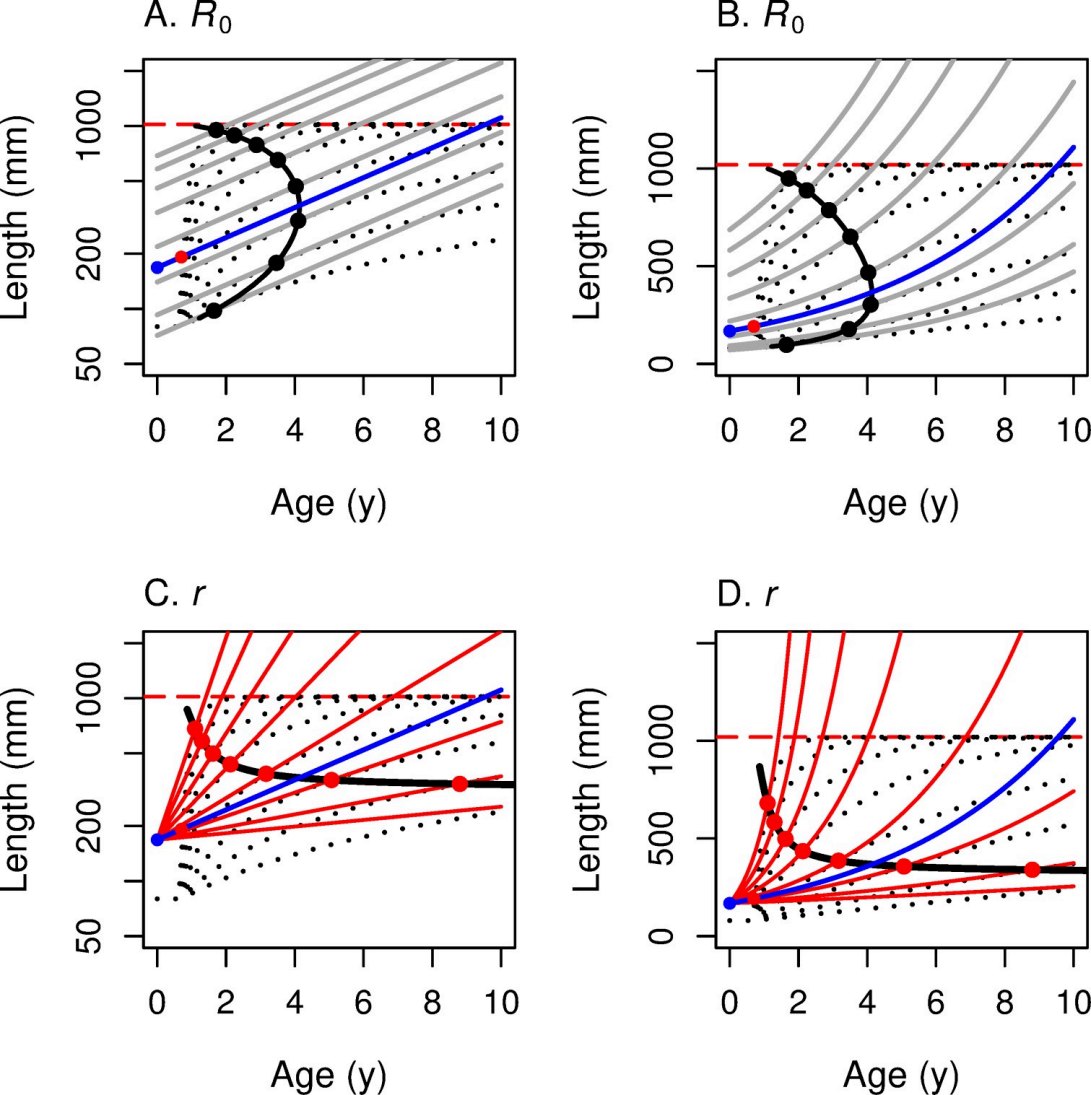
These panels illustrate the construction of maturation reaction norms (MRNs) for von Bertalanffy growth. This figure corresponds to Figs 2 and 3 for linear growth. *R*_0_ is the underlying fitness function for the top two panels (A, B); *r* is the underlying fitness function for the bottom two (C, D). The *y*-axis corresponds to log-length in the two panels on the left (A, C) and to linear length in the two panels on the right (B, D). The graphs of the von Bertalanffy growth curves are represented by black dotted lines in all 4 panels. The iso-fitness curves (from Fig 1) are solid grey lines in the top 2 panels and solid red lines in the bottom two. For each growth curve, one finds the highest iso-fitness curve that intersects it. The locus of such points, as growth varies, traces out the MRN (solid black curve). In the top two panels the MRN(*R*_0_) is a vertical line bowed to the right; in the bottom two panels the MRN(*r*) is mostly horizontal. The blue curve marks the *R*_0_ = 1 or *r* = 0 iso-fitness curve.

We put these graphs together to construct the graphs of the reaction norms. For each von Bertalanffy growth curve, we find the highest *R*_0_ level curve (Fig 1) that touches it. Fig 6A presents the reaction norm for *R*_0_ in *α-*ln(*L*) space, and Fig 6B presents the graph for Eq (0.33) in *α-L* space. Its U-shape follows from the fact that

$$\frac{d\alpha *}{dL*}=\frac{d\alpha *}{dk}/\frac{dL*}{dk}.$$

Here we can see that if the growth resource is limited, one should mature earlier if the resource decreases even more; on the other hand, it makes sense evolutionarily to take advantage of a high growth rate (plentiful resource) by spawning sooner rather than later.

#### *r* with von Bertalanffy growth

We next examine semelparous species with von Bertalanffy growth and fitness function *r*. Recall the optimization criterion (0.22). As we saw in Fig 1A and 1B, the level curves of *r* are straight lines through a common point in age/log-length space and a family of exponential curves from a common point in age/length space. For a given growth curve, we find the age at which the growth curve is tangent to the highest level set of *r* that it can reach.

Substituting the von Bertalanffy growth curve from (0.32) into the optimization criterion (0.22) for *r* yields the following:

$$\begin{array}{l} L\left(-\ln L_{\alpha 0}+\ln L\right)=\alpha L_{\infty }Cke^{-k\left(\alpha -y\right)}\\ \;=\alpha k\left(L_{\infty }-L\right) \end{array}$$ (0.35)

There is no simple analytical solution, but this expression can be solved numerically.

One has to solve (0.32) and (0.35) for α* and L* in terms of *k* to derive the maturation reaction norm MRN(*r*). In Fig 6C and 6D we present a computer solution to this system and present the geometric picture analogous to that of Fig 6A and 6B. As for the case with linear growth, the maturation reaction norms for *r* are close to horizontal curves so that optimal maturation age changes dramatically as *k* decreases, but the optimal maturation size does not change much as *k* changes.

#### Comparing maturation reaction norms for *R*_0_ versus *r* for von Bertalanffy growth

**Fig 7:**
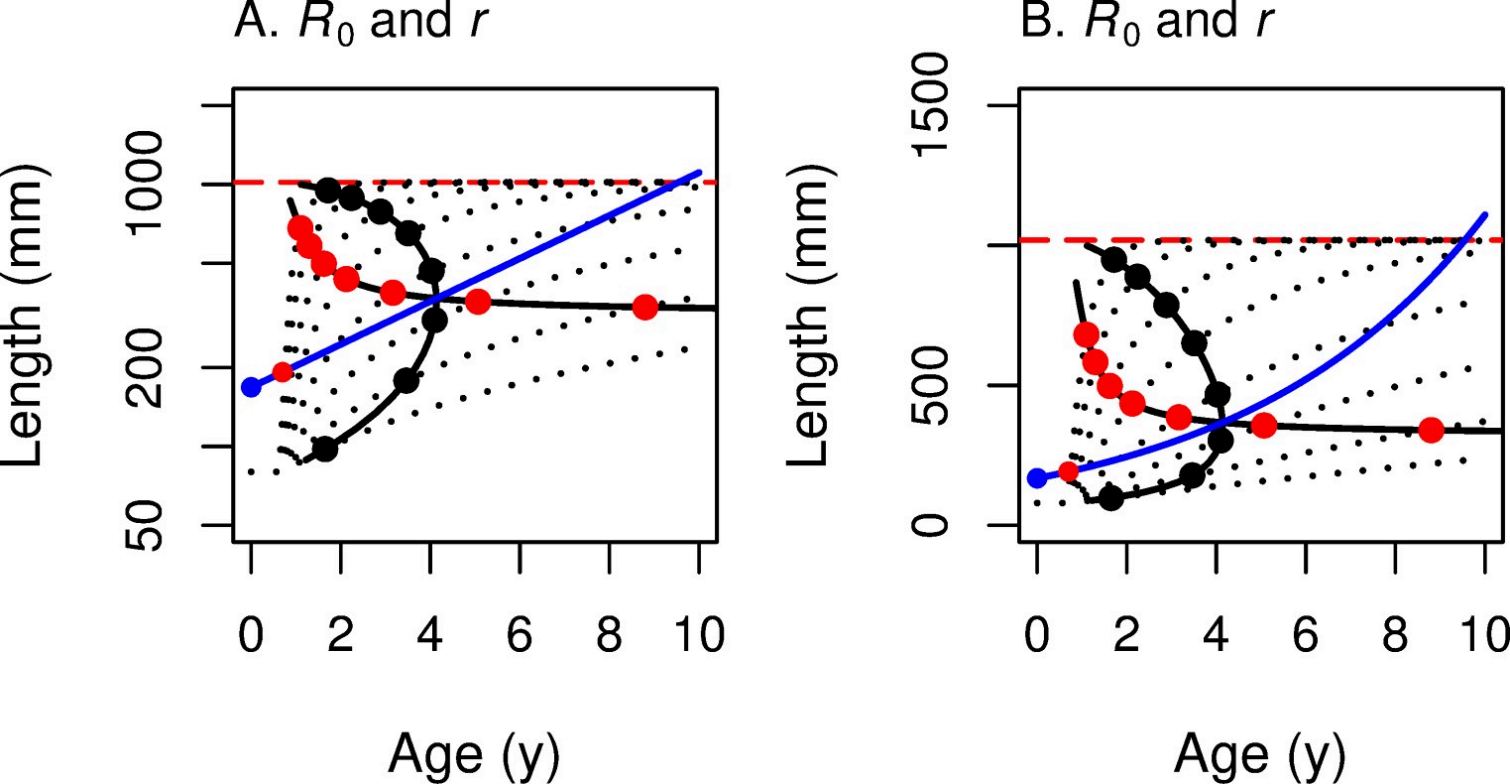
Growth trajectories with maturation reaction norms maximizing *r* (horizontal line) and *R*_0_ (bowed vertical line). This figure summarizes for von Bertalanffy growth trajectories (dotted lines) what Fig 4 summarized for linear growth; red dots show where *r* is maximized and black dots show where *R*_0_ is maximized along each growth trajectory. (A) With log-length on the vertical axis, the underlying iso-fitness curves are straight lines (shown only as the blue line that corresponds to *R*_0_ = 1 or *r* = 0; see Fig 6A and 6C). (B) With linear-length on the vertical axis, the underlying iso-fitness lines are curved (blue line corresponds to *R*_0_ = 1 or *r* = 0; see Fig 6B and 6D). In both panels, the reaction norm for fitness function *R*_0_ is bowed out to the right (optimal maturation *age* stays in a narrow range as growth rate varies), while the reaction norm for fitness function *r* is mostly horizontal (optimal maturation *size* changes little as growth rate varies).

In Fig 7, we plot the numerical solution of MRN(*R*_0_) and MRN(r) for a given set of parameters for von Bertalanffy growth, the analog of Fig 4 for linear growth. As expected, these two MRNs intersect where *R*_0_ = 1 and *r* = 0. That is, when the rate of change (of offspring per capita per year) for this lineage is 0, the optimal age and size for maximizing *r* are the *same* as the optimal age and size for maximizing *R*_0_. When this lineage is increasing (*R*_0_ > 1 and *r* > 0), then the optimal age and size for maximizing *r* are *less than* the optimal age and size for maximizing *R*_0_. As one moves along the highest growth trajectory in Fig 7, one first encounters the red circle indicating MRN(*r*) for von Bertalanffy growth. Farther along the same growth trajectory one then encounters the black circle of MRN(*R*_0_), indicating maturation at a later age and a larger size. The opposite occurs when the lineage is decreasing (*R*_0_ < 1 and *r* < 0): the age and size for maximizing *R*_0_ occurs earlier than the age and size for maximizing *r*. As for the case with linear growth (Fig 4), this figure for von Bertalanffy growth (Fig 7) shows a graphical explanation of the analytical result presented in (0.11). At extremely high growth rates, optimal size approaches the asymptotic size (indicated by the horizontal red dashed line in Fig 7).

As the population growth rate declines below the rate that would allow *R*_0_ = 1 and *r* = 0, the MRN(*r*) indicates that the *age* that maximizes *r* will increase rapidly, with little change in optimal size. In contrast, the MRN(*R*_0_) indicates that the age that maximizes *R*_0_ will change, first increasing, then decreasing, while optimal length decreases monotonically.

For linear growth, MRN(*r*) is horizontal, while MRN(*R*_0_) is vertical in age-length space (Fig 4). For von Bertalanffy growth, these patterns persist, especially for decreasing populations (*R*_0_ < 1) (Fig 7). A large difference between the MRNs occurs at very low von Bertalanffy growth rates, where MRN(*r*) indicates a very old age at maturity, whereas MRN(*R*_0_) indicates that optimal maturation will occur early at a small size; growth is so slow that waiting longer to mature would result in very small increases in fertility at the cost of much greater risk of death.

### Maturation reaction norms as the instantaneous mortality rate *z* changes

Up to this point, we have examined reaction norms for maturation age and size, *α* and *L*, as the underlying growth rate *k* changes. Geometrically, we expressed the fitness functions in terms of *α* and *L;* and for each growth curve with growth parameter *k*, we computed the highest fitness isocline that touched that growth curve. We turn now from varying *k* to varying the underlying instantaneous mortality rate *z*. Intuitively, if *k* measures the availability of a growth resource, like food supply or nurturing environment, then *z* measures the impact of predation or environmental hazards. We use a similar geometric process to take into account changes in *z*. However, *z* appears only in the expressions for survivorship in the fitness functions *r* and *R*_0_, not in the expressions for the growth curves. As we noted earlier, when viewed in *α*-ln(*L*) space (Fig 1A and 1C), changes in *z* lead to changes in the common slope (z/*b*) of the level sets of *R*_0_ (0.15), to changes in the various slopes ((*r* + *z*)/*b*) of the level lines of *r* (0.19), and, for *y* > 0, to changes in the common intercept of the concurrent level lines of *r* (0.22).

**Fig 8:**
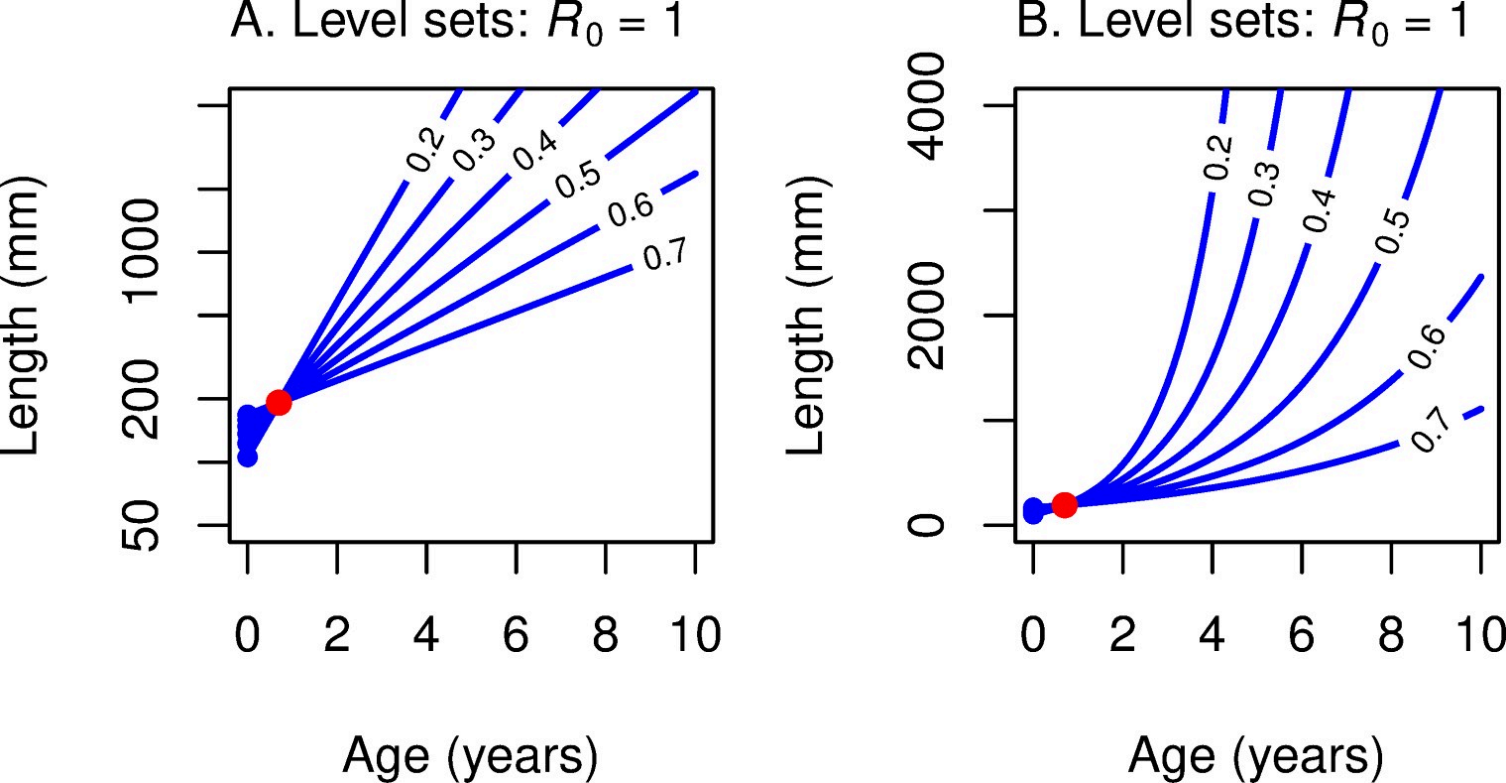
Level sets for *R*_0_ = 1, *r* = 0 in age-length space, by annual survival. (A) With log-length on the vertical axis, iso-fitness lines are straight. (B) With linear-length on the vertical axis, iso-fitness lines are curved. As annual survival probability increases from *s* = exp(-*z*) = 0.2 to 0.7, these level sets rotate clockwise about the red point (*y*, *L*_*y*_) and the age-0 intercept (0, *L*_α0_) increases. (See the blue curves in Fig 1.).

**Fig 9:**
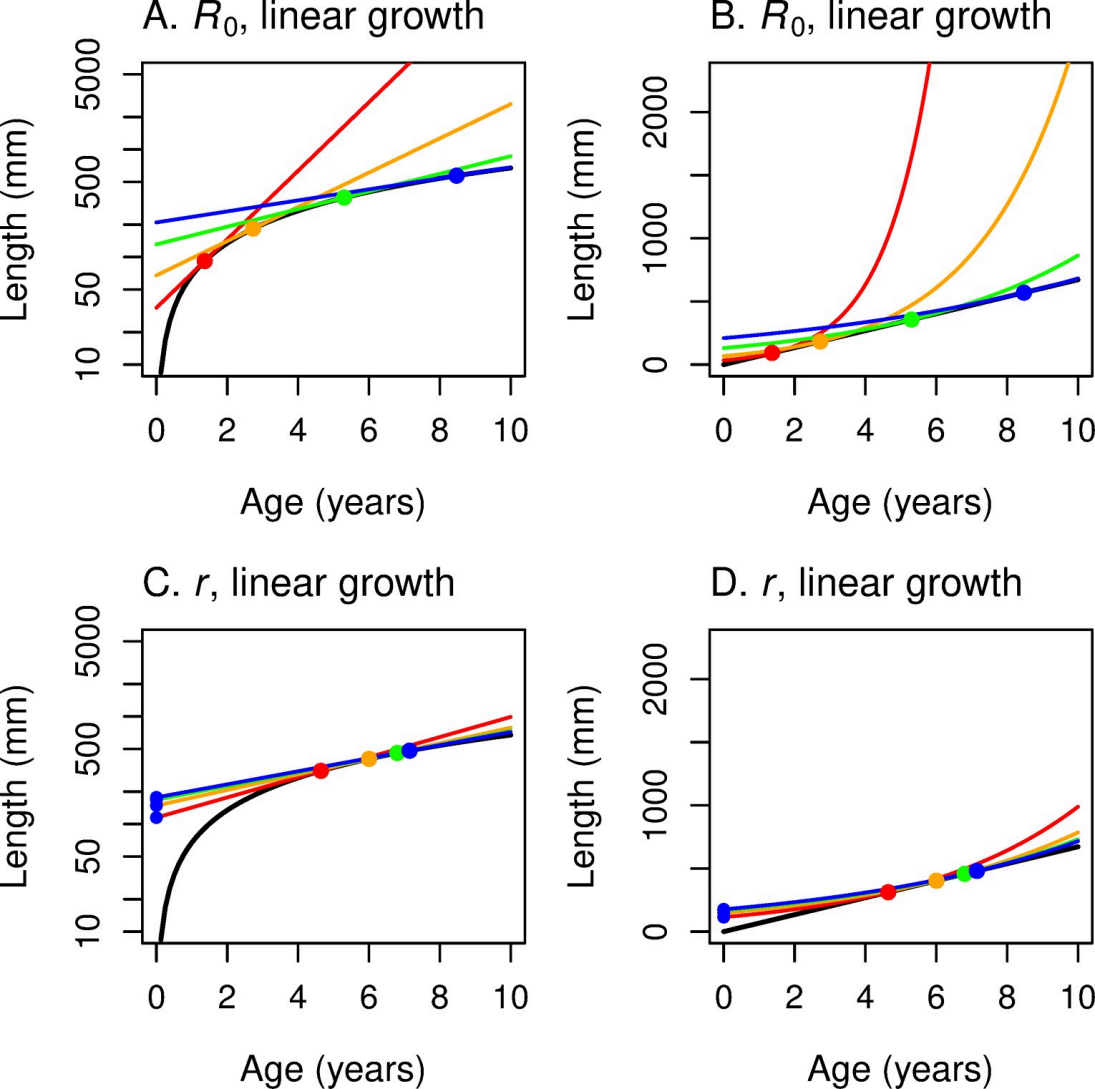
Given a linear growth trajectory, increasing survival increases optimal maturation age and size. As annual survival *s* increases, the growth curve is unchanged, but the iso-fitness curves become less steep and the age-0 intercepts increase (see Fig 8), so optimal age and length increase along the growth curve. From this point of view the reaction norm curve is the same as the growth curve. Colored lines show level sets of fitness function *R*_0_ in (A, B) and level sets of *r* in (C, D); corresponding colored points show optimal age and length to maximize the fitness function for each level of annual survival rate *s* (red: 0.25, orange: 0.50, green: 0.70, blue: 0.80). This linear growth trajectory has *k* = 266.2 mm/yr, *c* = 0. (A, C) With log-length on the vertical axis, iso-fitness lines are straight. (B, D) With linear length on the vertical axis, the linear growth curve is a straight line, but iso-fitness lines are curved.

**Fig 10:**
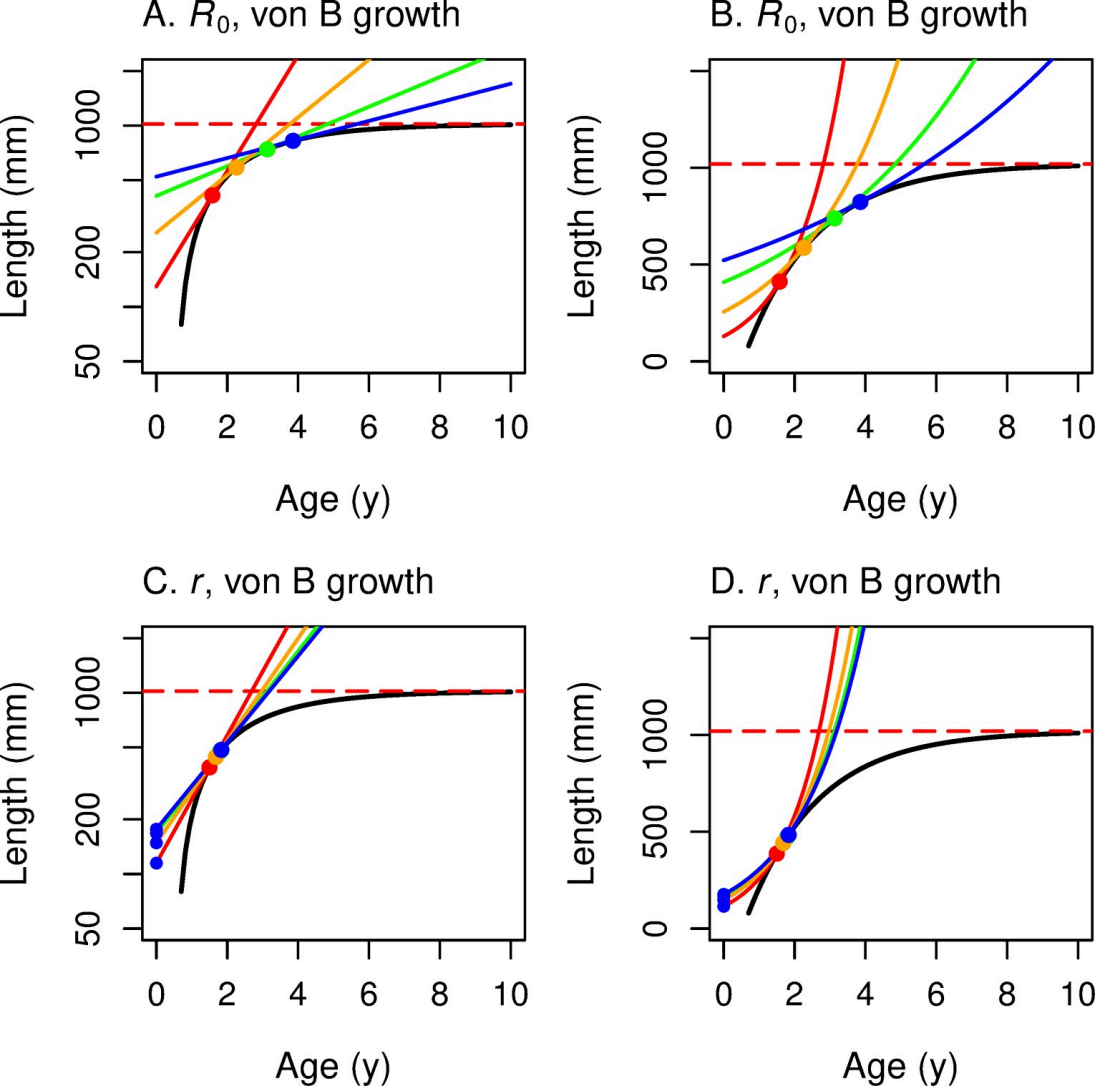
Given a von Bertalanffy growth trajectory, increasing survival increases optimal maturation age and size. As annual survival *s* increases (red: 0.25, orange: 0.50, green: 0.70, blue: 0.80), the growth curve is unchanged but the iso-fitness curves become less steep and the age-0 intercepts increase, so optimal age and length increase along the growth curve. From this point of view the reaction norm curve is the same as the growth curve. (A, C) With log-length on the vertical axis, iso-fitness lines are straight. (B, D) With linear length on the vertical axis, iso-fitness lines are curved. The horizontal red dashed line represents the asymptotic length *L*_∞_ for von Bertalanffy growth, with *k* = 0.4973 yr^-1^, *L*_∞_ = 1019 mm, *y* = 0.701 yr, and *L*_*y*_ = 80 mm.

We fix growth parameter *k*, which means we fix the underlying growth curve. For maximizing *r*, recall that for each fixed value of *z*, the level sets of *r* in age-log(length) space form a “spray” of lines with common intercept point (0.14) (Fig 8A and 8B). As *z* increases, this intercept decreases (given *y* > 0) and the slope of the *r* = 0 line increases (Fig 8). As *z* increases, the *r*-level line that is tangent to the growth curve moves down and to the left (Fig 9C and 9D for linear growth; Fig 10C and 10D for von Bertalanffy growth). It follows that the *z*-maturation reaction norms in age-log(length) space are simply the growth curves, with optimal maturation age and size both decreasing as mortality rate *z* increases. Note that if *y* = 0, then the intercept for the level sets of *r* does not change with *z*. In this case, the *r*-level set that is tangent to the growth curve has the same point of tangency, so that the optimal size and age of maturation are independent of *z*.

A similar story holds for fitness function *R*_0_; increases in *z* lead to increases in the slope of the *R*_0_ level lines, which in turn implies that the optimizing tangency occurs below and to the left of the original tangency. Once again, the growth curve itself is the maturation reaction norm as *z* changes. Optimal maturation age and size both decrease as mortality rate *z* increases (Fig 9A and 9B for linear growth; Fig 10A and 10B for van Bertalanffy growth).

Compare Fig 9B with Fig 1 in [4], derived from simulations because the authors are treating the iteroparous case and hold *b* = 3.

### Two-dimensional reaction norms

**Fig 11:**
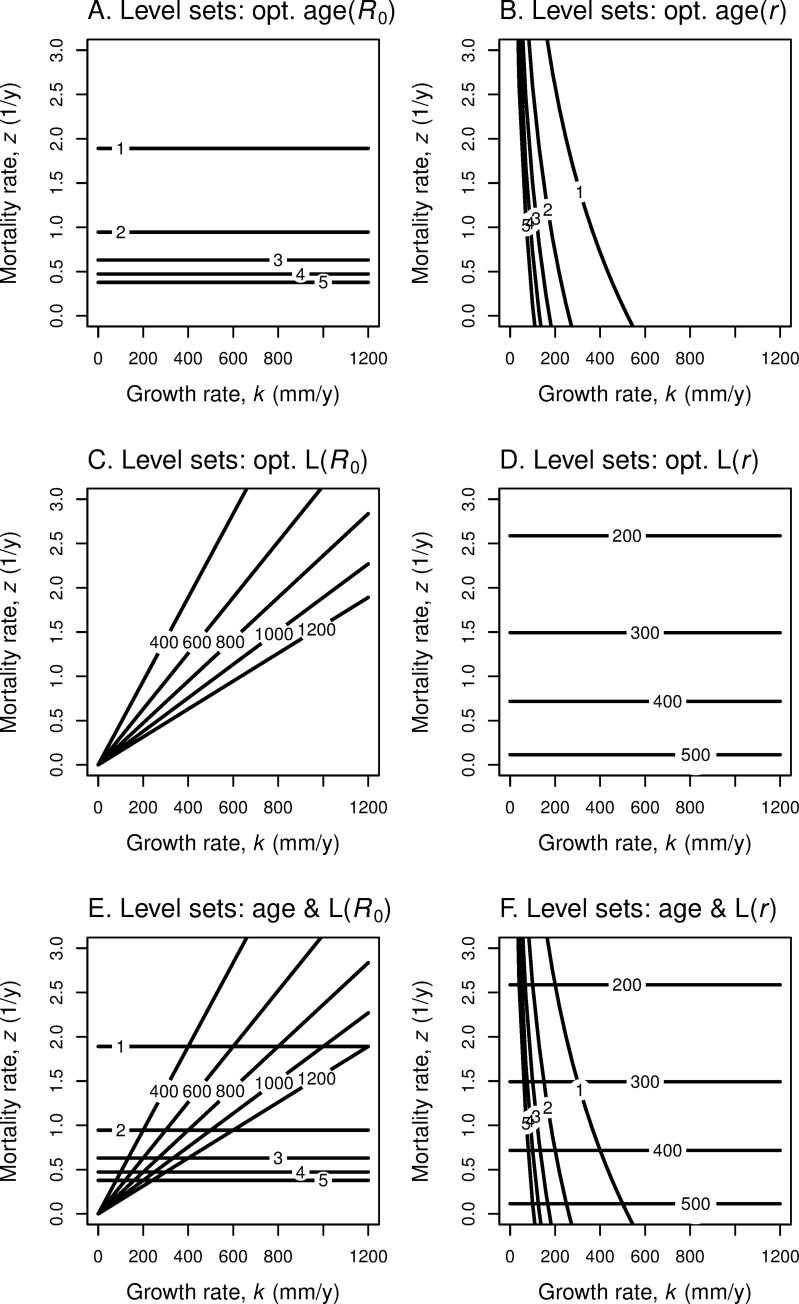
Level sets of optimal age and length as functions of growth rate and mortality rate. The underlying growth is linear. For each combination of growth rate *k* and mortality rate *z* and underlying fitness function, there is an optimal age *α* of maturity and optimal length *L*. (A) Level sets of optimal age for fitness function *R*_0_, with the age listed on each level set; (B) Level sets of optimal age for fitness function *r*; (C) Level sets of optimal length for fitness function *R*_0_, with the length listed on each level set; (D) Level sets of optimal length for fitness function *r*; (E) combines information from (A) and (C); (F) combines information from (B) and (D). Optimal age is insensitive to *k* for *R*_0_; optimal length is insensitive to *k* for *r*.

We now ask what happens to optimal age and size at maturation as *both k* and *z* change. As our discussions in the previous two subsections indicate, the same equations apply to maximizing with respect to *k* and with respect to *z*. For fitness function *R*_0_, Eq (0.33) present the optimal *α* and the optimal *L* for each choice of *k* and *z*. Fig 11 displays the level sets of optimal *α* and *L* respectively in (*k*,*z*)-space for linear growth, for fitness function *R*_0_ and also for fitness function *r*.

For the case of linear growth, we see (Fig 11) that if *k* changes but not *z*, optimal age *α* does not change for fitness *R*_0_ but it increases monotonically for fitness *r*. If mortality rate *z* increases, but *k* stays constant, optimal *α* decreases under *R*_0_ and also decreases under *r*. If both *k* and *z* increase, α decreases under *R*_0_, but changes relatively more slowly under *r*.

**Fig 12:**
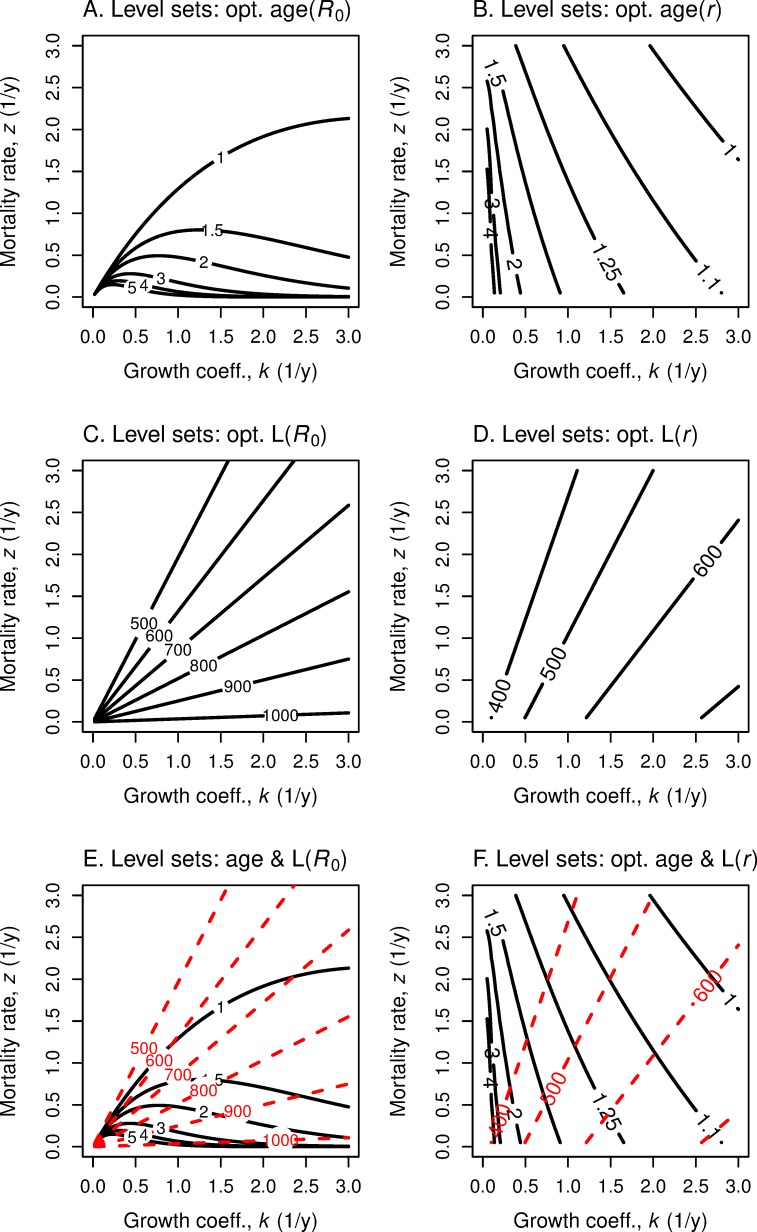
Level sets of optimal age and length as functions of growth rate and mortality rate. These panels repeat the analyses in Fig 11 but use von Bertalanffy growth.

The level sets for optimal maturation age are different for linear growth (Fig 11) and von Bertalanffy growth (Fig 12). Also, the optimal size level sets differ for the two growth functions (Fig 11, Fig 12). For von Bertalanffy growth, to maintain the same optimal size for fitness *r*, an increase in *k* requires a compensatory increase in *z* (Fig 12D). For linear growth, the optimal maturation size for fitness *r* is independent of the growth rate *k* but strongly influenced by mortality rate *z* (Fig 11D).

To get the full picture for how optimal age and size vary as the underlying parameters *k* and *z* vary, one can create a *four-dimensional* reaction norm graph by superimposing the optimal age graphs over the optimal size graphs (Fig 11E and 11F and Fig 12E and 12F).

Fig 12 allows one to sketch reaction norms when there is an underlying relationship between survivorship and growth. For example, in their treatment of iteroparous species, Perrin and Rubin [4] used simulations to draw different reaction norms when survivorship and growth are positively correlated (their Fig 3) or negatively correlated (their Fig 4). In the latter case, their reaction norms are “dome-shaped,” like those that Alm [36] found in his experimental work with several fish species.

## Optimal fitness trade-offs as *k* and *z* change

Finally, we relate optimal fitness to values of the growth parameter and mortality rate. In this way, we can discuss the trade-off between increasing growth rate *k* (via more resources, like food) and increasing risk of mortality (say, via more predators or harsher environment). All species face this risk-reward dilemma: trading off the possibility of harm or death in order to achieve a more valuable resource. Here we use growth rate *k* to measure resource acquisition and instantaneous mortality rate *z* to measure “risk.” For each (*k*,*z*) pair we have computed the optimal age α and size *L* of maturation and for each such pair (*α*, *L*) we can compute fitness *r* and fitness *R*_0_. This gives us optimal fitness as a function of *k* and *z*.

For the reproductive rate *R*_0_, we substitute α and *L* from Eqs (0.33) into expression (0.12) for *R*_0_:

$$\begin{array}{l} R_{0}=q\cdot l_{y}\cdot e^{-z(\alpha -y)}A{\left(L_{\alpha }\right)}^{b}\\ \;=q\cdot l_{y}{\left(C\left(\frac{bk+z}{z}\right)\right)}^{-\frac{z}{k}}A{\left(L_{\infty }\right)}^{b}{\left(1-\frac{z}{bk+z}\right)}^{b}\\ \;=q\cdot l_{y}\cdot A{\left(L_{\infty }\right)}^{b}{\left(C\left(b\frac{k}{z}+1\right)\right)}^{-\frac{z}{k}}{}^{}{\left(\frac{1}{1+\frac{z}{bk}}\right)}^{b} \end{array}$$ (0.36)

**Fig 13:**
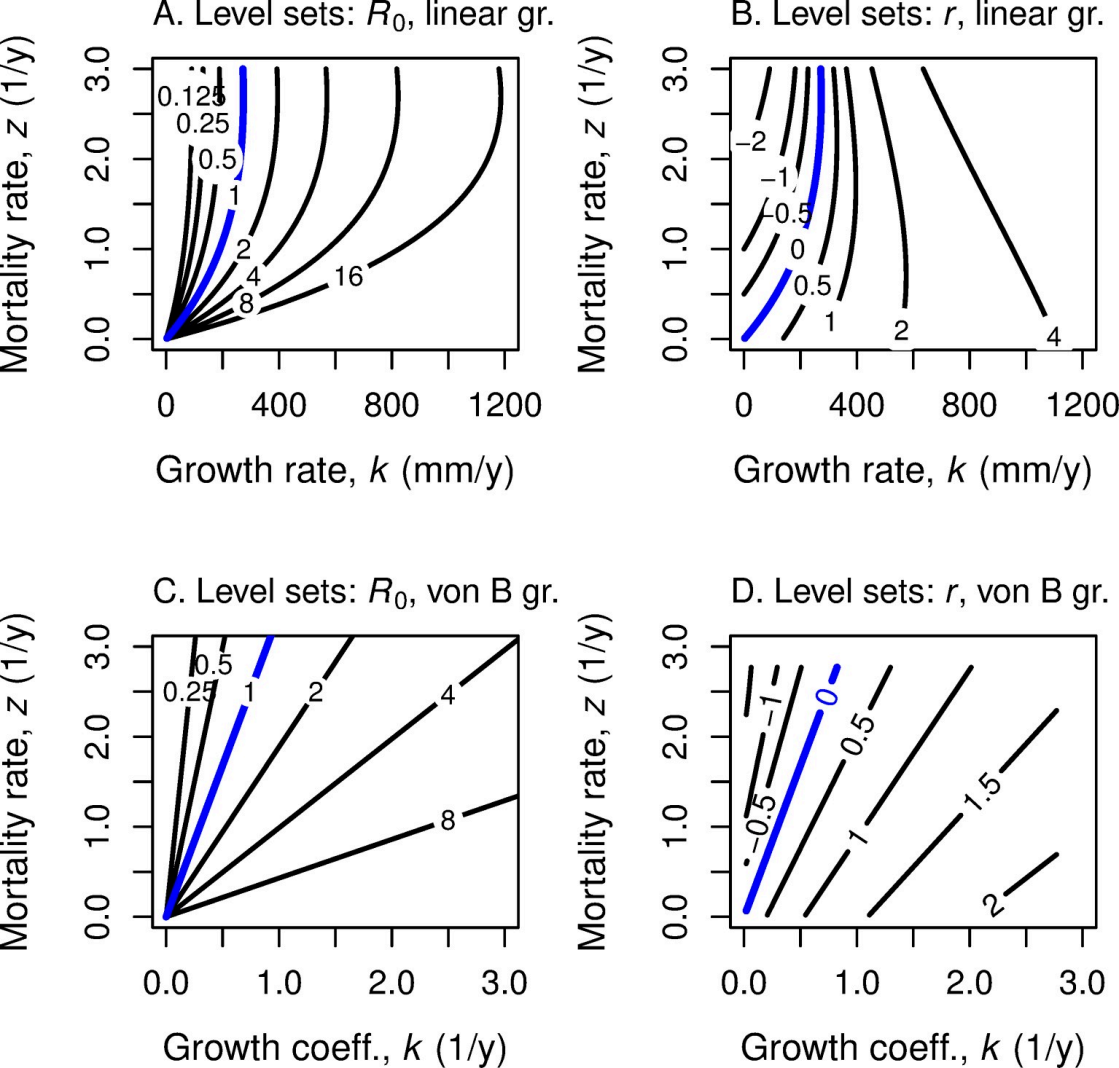
Level sets of fitness as functions of growth rate and mortality rate. Level sets of fitness as *R*_0_ (A, C) and fitness as *r* (B, D) in relation to mortality rate *z* and growth coefficient *k*, for linear growth (A, B) and for von Bertalanffy growth (C, D). Combinations of growth rate *k* and mortality rate *z* in the region below and to the right of the blue line have *R*_0_ > 1 and *r* > 0 and thus indicate the lineage is increasing. Such a figure helps in the evaluation of the trade-offs, say, in the search for a more substantial but riskier food source.

The first line of (0.36) follows from (0.12); the second line from (0.33). Note that *k* and *z* only occur in the ratio *k*/*z*. Thus, the *R*_0_ level curves are straight lines from the origin in (*k*,*z*) space for von Bertalanffy growth (Fig 13C), in contrast to the curved lines for linear growth (Fig 13A).

The corresponding level curves of fitness *r* require a numerical approach. Note that for linear growth, the level sets of *r* are more “parallel” (Fig 13B) than are those of *R*_0_. For von Bertalanffy growth, the level sets of *r* do not arise from the *k-z* origin (Fig 13D). For *r* the tradeoff between improved resources for growth *k* and the risk in attaining those resources does not depend much on current fitness. However, for *R*_0_ the lower one’s fitness level, the more risk one is willing to trade off for the resources for higher *k*.

### Comparison with data

#### Naturalized Chinook Salmon in Lake Michigan

It is natural to ask whether changes in growth rate cause Chinook Salmon in Lake Michigan to modify their age at maturity (in line with *r*) or their size at maturity (in line with *R*_0_). We present some preliminary data here; we will compile a more complete empirical analysis in a follow-up report.

**Fig 14:**
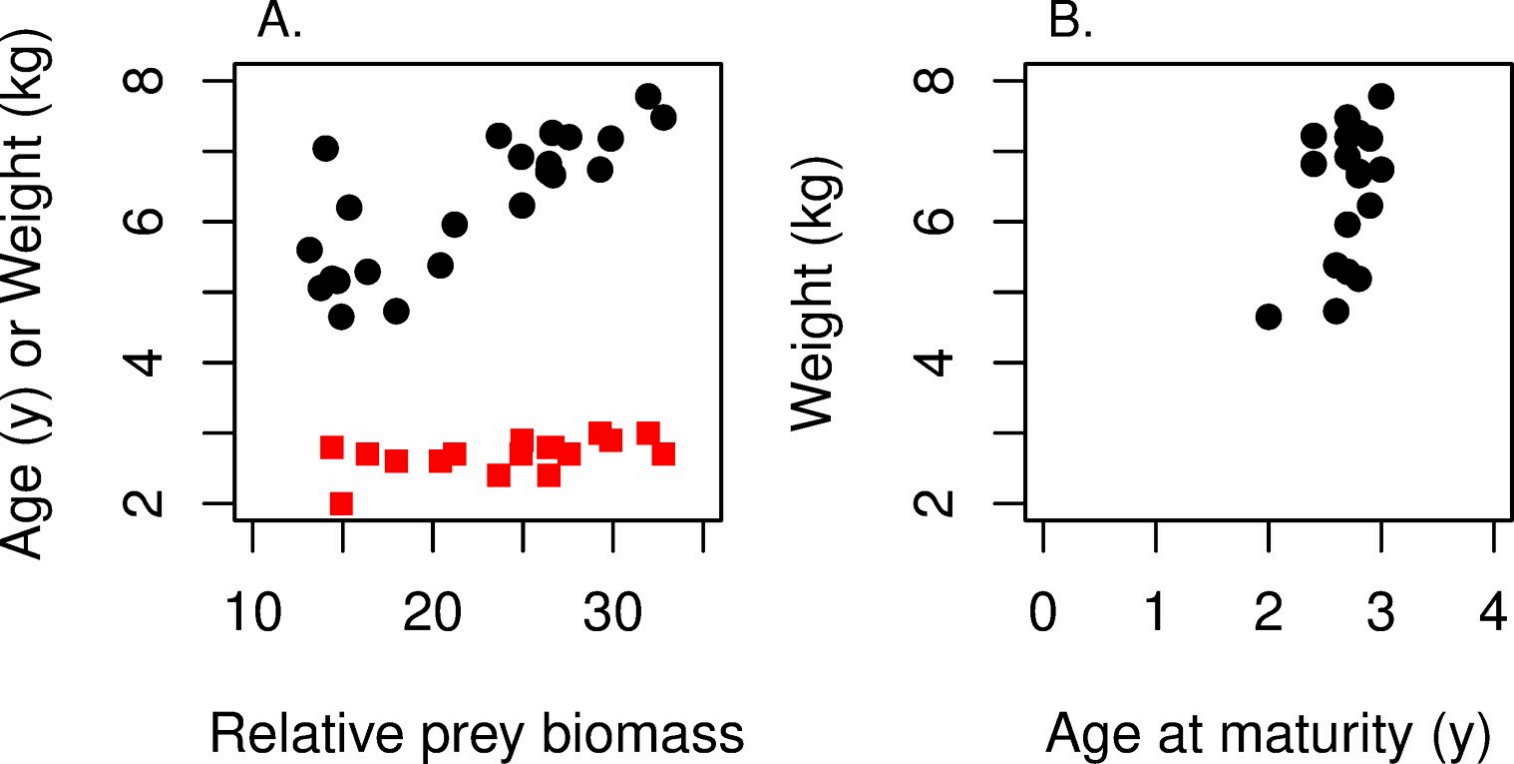
Weight and age at maturity for female Chinook Salmon in Lake Michigan. (A) Weight at maturity in kg (black circles) and age at maturity in years (red squares) for female Chinook Salmon in Lake Michigan each year for the period 1991–2008 [37] in relation to the relative prey biomass (tons of Alewife/tons of Chinook Salmon). (B) Weight at maturity relative to age of maturity for these same points. In both panels, we see small changes in age at maturity and relatively large swings in size at maturity.

A major factor affecting fish growth rate is the availability of preferred foods. The preferred food of Lake Michigan Chinook is the Alewife (*Alosa pseudoharengus*), a fish related to herring. The relative biomass of Alewives (tons of Alewives per ton of Chinook Salmon) in Lake Michigan varied by a factor of two during the period 1991–2008 [37]. Fig 14 summarizes these data. For any prey biomass, the female age at maturity mostly hovers between 2.4 and 3.0 years, while the female weight at maturity increases with relative prey biomass roughly from 5 to 8 kg (Fig 14A). (For salmonids that spawn at a single time each year, roughly October 1 for the Chinook Salmon in Lake Michigan, an age of maturity of 2.5 is interpreted as 50% of the females maturing at age 2 and 50% at age 3.) Fig 14B plots age at maturity versus weight at maturity for these 18 points; it presents a relatively constant age and an increasing size. We note that the data in Fig 14 broadly represent Lake Michigan Chinook Salmon in that they come from an empirical study [37] of multiple tributaries over many years.

It is natural to ask how the data points in Fig 14B relate to the MRNs in Fig 4 (linear growth) and Fig 7 (von Bertalanffy growth). To draw the relevant MRNs we used the values for the parameters we estimated for Lake Michigan Chinook Salmon (S1 Appendix, especially Appendix Table B). We then drew the MRNs for *r* and *R*_0_ by letting growth rate *k* vary.

First consider linear growth. Many authors [4,23–28] work with linear growth for fish; some even present empirical evidence of linear growth, at least prematuration. The points in Fig 14B are clearly more consistent with the vertical MRN(*R*_0_) than with the horizontal MRN(*r*) in Fig 4.

**Fig 15:**
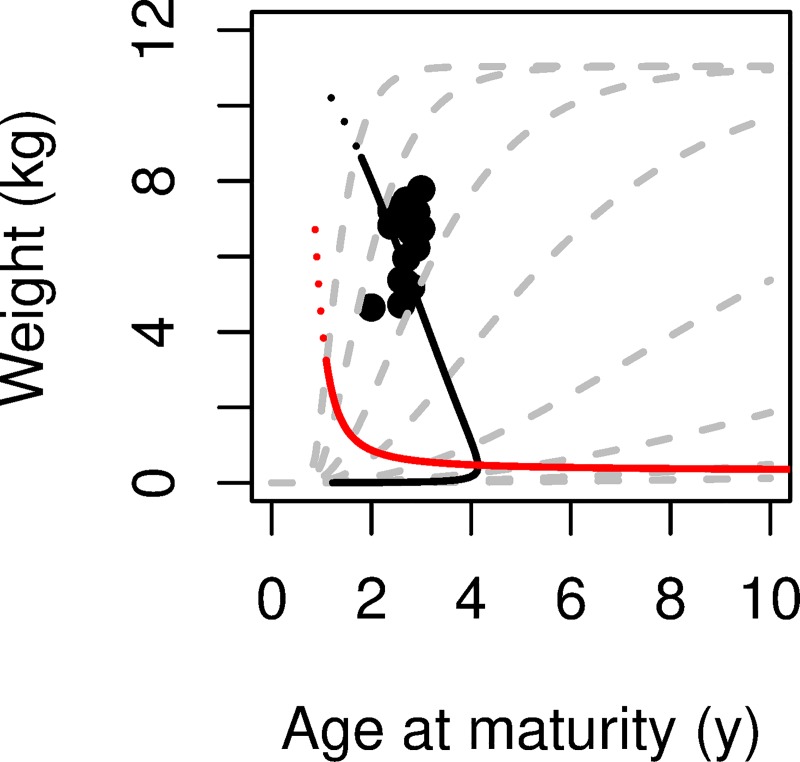
Weight and age at maturity for female Chinook Salmon in Lake Michigan, with growth trajectories and maturation reaction norms for *R*_0_ and *r*. Data points from Fig 14B for the period 1991–2008 [37] are shown with growth trajectories (dashed gray lines), MRN(*R*_0_) (black line) and MRN(*r*) (red line) from Fig 7B, converting length to weight [38].

Turning to von Bertalanffy growth, we overlay in Fig 15 the maturation size and age data from Fig 14B with the von Bertalanffy growth trajectories and MRNs from Fig 7B, converting length to weight [38]. The second-highest and third-highest growth trajectories (with growth coefficients of 0.64 and 1.28 y^-1^) bound the almost two-fold variation in mean weight at maturity. Within this range of growth rates both MRNs show an increase in maturation age with a decrease in weight at maturity. The MRN(*R*_0_) goes through the range of data points. In striking contrast, the MRN(*r*) for the same range of growth rates predicts a weight at maturity about three-fold smaller and an age at maturity about one year younger than the data. The data are more consistent with MRN(*R*_0_) than MRN(*r*).

### Hatchery-reared Chinook Salmon

A comparison of hatchery-reared salmon with wild salmon also points to *R*_0_ as the more likely underlying fitness function. For maximizing *r* (but not for maximizing *R*_0_), the egg-to-smolt survival affects the optimal maturation age and length. (Compare (0.23) and (0.24).) Thus, if *r* is the underlying fitness function, changes in this early survival will change the optimal maturation age and length.

Under natural conditions this survival fraction (which we denote as *l*_*y*_) may be low. For naturalized Chinook Salmon in Lake Michigan we use an estimate of *l*_*y*_ = 0.00953 (see Table B in S1 Appendix). In hatcheries, this egg-to-smolt survival is much greater; that is the purpose of hatcheries. Salmon hatcheries in British Columbia, Canada, have egg-to-smolt survivals of 0.70–0.80 [39]. Hatcheries on the Lower Snake River in Idaho, for the 2005 Chinook Salmon brood year, had a reported egg-to-release (at smolt) survival of 0.813 [40].

If we use *R*_0_ as the underlying fitness function and use our nominal values for von Bertalanffy growth and other parameters (see Table B in S1 Appendix), this results in an optimal maturation age of 3.1 years after fertilization and an optimal maturation length of 739 mm, with an expected fecundity of 2,012 female eggs. The resulting value of *R*_0_ is 5.37 females per female per generation. These values are expected to be the same for wild and hatchery Chinook Salmon, because the parameter *l*_*y*_ does not influence the calculations for *R*_0_.

If we use *r* as the underlying fitness function and continue using our nominal values for von Bertalanffy growth and the other parameters, then *l*_*y*_ = 0.00953 results in an optimal maturation age of 1.8 years and an optimal maturation length of 472 mm, with an expected fecundity of 861 female eggs. The hatchery value of *l*_*y*_ = 0.813 produces dramatically different results; the optimal maturation age becomes 0.89 years, the optimal maturation length becomes 163 mm, with an expected fecundity of 115 female eggs (with 0.813 surviving to smolt!). The growth rate of the lineage would increase from *r* = 0.735 to *r* = 4.59, corresponding to the annual growth multiplier λ = e^*r*^ increasing from λ = 2.1 to λ = 98. However, the observation is that stocked hatchery-reared fish tend to mature at the *same* age and length as wild (or naturalized) fish [37]. This strongly suggests that the salmon are not maximizing *r*, at least not under these conditions.

## Discussion

### Overview

Optimal age and size at maturity depend on many life history parameters, including early-age survivorship (our *l*_*y*_), juvenile survival (our *z*), and fertility as a function of body size (*A L*^*b*^). These need to be estimated in order to compute optimal life history characteristics. Once functions are specified for survivorship as a function of age, fertility as a function of size, and growth as a function of age, then one can write fitness (as *R*_0_ and as *r*) as a function of age and size.

Several authors have correctly pointed out that maximizing *r* and maximizing *R*_0_ yield the same optimal life history traits for the case when *r* = 0 and *R*_0_ = 1. However, as environmental conditions change or vary, the growth rate or mortality rate is likely to change, leading to changes in *r* and *R*_0_. We have shown how the optimal age and length at maturation would change for each fitness function given such potential changes in *r* and *R*_0_.

The case of linear growth in length clearly shows the differences in maturation reaction norms between these two classic fitness functions. Furthermore, the linear-growth case also helps interpret the variation on this theme seen in the MRNs for von Bertalanffy growth.

Given our assumptions for survivorship, fertility, and growth, we show the following, for linear growth (Fig 4). (1) MRN(*R*_0_) is a straight *vertical* line in age-length space, so that optimal *age* at maturation is constant despite changes in growth rate. The optimal age is proportional to the length exponent for fertility *b* and inversely proportional to mortality rate *z*; (2) MRN(*r*) is a straight *horizontal* line in age-length space, so that optimal *size* at maturation is constant despite changes in growth rate. The optimal size is *L*_α_ = e·*L*_α0_, where *L*_α0_ = (*l*_*y*_ e ^*zy*^ *A*)^(-1/b)^; that is, related to survivorship to smolting *l*_*y*_, age at smolting *y*, juvenile mortality rate after smolting *z*, the length coefficient for fertility *A*, and the length exponent for fertility *b*. (3) The pattern in MRNs is similar for von Bertalanffy growth, but in this case the MRN lines are curved, not straight (Fig 7).

For semelparous species, both analytical and graphical analyses show that the optimal age of maturation for maximizing *r* is *younger* than the optimal age of maturation for maximizing *R*_0_, but only when *r* > 0; this *reverses* when *r* < 0. This difference is dramatic for linear growth (Fig 4), and is also apparent for von Bertalanffy growth (Fig 7). In fact, the analytical result applies for any increasing growth function.

We believe this is the first demonstration of this difference between these fitness functions. The graphical depiction of the two MRNs presented here explains this analytical result for the two fitness functions. Because the two MRN lines intersect (at *r* = 0 and *R*_0_ = 1), a growth trajectory that passes to the left and above the intersection point (so that *r* > 0) must first encounter MRN(*r*) and then MRN(*R*_0_). Conversely, a growth trajectory that passes below and to the right of the intersection point (so that *r* < 0) must first encounter MRN(*R*_0_) and then MRN(*r*). These differences between the fitness functions in optimal maturation age have a parallel in the differences in optimal maturation length, which may be of more interest to anglers and fisheries managers. Following along a particular growth trajectory that passes to the left and above the intersection point of the MRNs (so that *r* > 0), one can see that the optimal maturation length that maximizes *r* is smaller than the optimal maturation length that maximizes *R*_0_. Again, this reverses for growth trajectories that result in *r* < 0.

In the rest of this discussion section, we examine some of the choices we made in constructing our underlying models.

### Alternative functions for survivorship and fertility

Roff [1] and others [11,41] have used a simpler model of survivorship than we do (Eq 0.12), by assuming that much of early mortality is concentrated very shortly after hatch, followed by a constant mortality rate that applies to the subsequent juvenile period. Mathematically, their approach would simplify our equation for survivorship from *l*_*x*_ = *l*_*y*_ *e−*^*z (x-y)*^ to *l*_*x*_ = *p e* ^*−z x*^, setting our *y* = 0 and *l*_*y*_ = *p*. This simplification is not a good description for survival of salmon, because the early period in the natal stream can last months or years [42], so that *y* >> 0, and growth and mortality in the stream is much different than in the ocean or Great Lakes. However, Roff’s [1, page 184] model does give a result consistent with ours: with *r* as the fitness function, his model indicates that optimal maturation age depends on *p* but not *z*. Our model gives the same result, but only for the case with *y* = 0; in the more biologically realistic case of *y* > 0, our model indicates that both *p* and *z* influence optimal maturation age. With *R*_0_ as the fitness function, both our model and Roff’s [1] model give the result that optimal maturation age depends on *z* but not on *p*.

Skorping et al. [43] assume, as we do, that juvenile mortality rate *z* is constant, but they assume that fertility increases linearly with age. Their approach is equivalent to setting *b* = 1 in our fertility function (0.13): *m*_*x*_ *= A(L*_*x*_*)*^*b*^. Using *R*_0_ as the fitness function for their semelparous species, they find that the optimal age to mature is *1/z*. Our model for linear growth in length gives the optimal maturation age as *b*/*z*. Our model would reduce to the Skorping et al. [43] model for *b* = 1 and *y* = 0. [Skorping et al. note that their maturation age corresponds to the time when survivorship decreases to a constant value of 0.368 = e^−1^.]

Charnov and Berrigan [44] discuss the Beverton-Holt life-history invariants, *αz*, *z*/*k*, and *L*_α_/*L*_∞_, as do [5,8,35]. Charnov and Berrigan [44] point out that populations with the same values of *z*/*k* and *L*_α_/*L*_∞_ will have the same value of *αz*. The product *αz* as a constant arises in our approach, for one can show, using (0.33) and (0.34) that semelparous fish with von Bertalanffy growth that are maximizing *R*_0_ will have

$$\left(\alpha -y\right)z=\left(\frac{z}{k}\right)\left[\ln(C)-\ln\left(1-\frac{L_{\alpha }}{L_{\infty }}\right)\right],$$ (0.37)

with the invariants appearing as ratios or products. This equation suggests that the relationships for salmon should be examined using “lake age” or “ocean age” (*α* –*y*) rather than total age *α*. Semelparous fish with linear growth in length that are maximizing *R*_0_ will have *α* = *b*/*z*, so that *αz* equals the fertility parameter *b*. Charnov and Berrigan [44] have the *y* and *C* in (0.37) equal to 0 and 1, respectively. Perhaps some of the observed variation in the “invariants” among taxa [44] is due to differences in early life history that would translate to differences in *y* and *C*.

Maximizing fitness *R*_0_ in fisheries models of growth and survival, Jensen [8] obtained theoretical values of the Beverton and Holt invariants. He then compared his theoretical estimates with regression-based estimates using data from 19 freshwater fish populations. The values of the invariant constants obtained from regression across species agreed fairly closely with the theoretical values of the constants, suggesting that the life history invariants result from fundamental ecological relations among the parameters. Our Eqs (0.34), (0.36) and (0.39) show such relationships, and include additional parameters (*y*, *C*, and *b*) that may be helpful in accounting for some of the observed differences in the “invariants” among taxa.

### Parameters needed for calculation

In order to test models for age and length at maturity, one needs, of course, observed *values* of age and length at maturity. One also needs estimates of other parameters to calculate predicted values, including parameters for size as a function of age, fecundity as a function of size, and mortality rate during the juvenile period. If one wants to calculate predictions based on maximizing *r*, then one also needs survivorship from spawning (or hatch) to the age at which juvenile mortality rate becomes constant. Comparison of expression (0.21) for optimal age and size under fitness *R*_0_ with the corresponding expression (0.22) for fitness *r* makes clear the need for more parameters when maximizing with *r*, especially parameters on early life survival. This difference may give *R*_0_ a slight advantage over *r* as the natural fitness function.

As we pointed out in the previous section, if one concentrates stream life history into one small interval near *t* = 0, or equivalently set *y* to 0, then mortality *z* drops out of the expressions for optimal age and size.

### Correlations in growth and mortality

Changes in growth rate are sometimes *negatively* correlated with changes in mortality rate. For example, slower growth might accompany higher mortality if food becomes less abundant and the chance of starvation or predation increases. Alternatively, growth rate may be *positively* correlated with mortality rate if environments with more abundant food also have higher risk of predation. Choice of such environments has been observed in the lab [45,46] and in the field [47]. Haugen et al. [47] present evidence that northern pike adjust the probability of moving between the two ends of Lake Windermere to equalize relative fitness–one basin with higher food production and higher risk of mortality, the other basin with lower food production and generally lower risk of mortality. The growth (hence fecundity) and mortality in both basins were strongly influenced by pike density. These pike adjust their dispersal probabilities with the result that densities are adjusted between the north and south ends of the lake resulting in roughly equal fitness in each end of the lake. This situation is illustrated in our Fig 13. Whether *r* or *R*_0_ is the underlying fitness function, Fig 13 displays iso-fitness curves that go from low growth/low mortality to high growth/high mortality diagonally across growth/mortality space—the situation where growth and mortality are positively correlated.

### Observed patterns in reaction norms

If fish experience slower growth rates, while other parameters remain the same, then there are three possible responses in terms of age at maturity (age could move earlier, remain the same, or move later) and three possible responses in terms of length at maturity (length could become smaller, remain the same, or become larger). Four of these nine combined responses are unlikely: mature at the same age and the same or larger size; or mature at an earlier age and the same or larger size. For example, suppose that under a regime of slower growth, fish matured at the same or larger size. Since it takes more time to reach this size in this new regime, age at maturity automatically increases.

Working only with fitness function *r*, Stearns and Koella ([3]; see also [50]) describe the five remaining patterns they found in their studies of how age and size at maturity respond to decreases in growth rate. “When organisms are forced to grow more slowly, they 1) mature later at a smaller size, 2) mature later at the same size, 3) mature later at a larger size, 4) mature earlier at a smaller size, or 5) mature at the same age at a smaller size” [3, p. 894]. Our maturation reaction norms can describe four of these five patterns, and the remaining pattern could be produced if decreases in growth rate were accompanied by decreases in mortality rate. More details are given in S7 Appendix.

As growth decreases to very low levels, our MRN(*R*_0_) specifies ever younger ages of maturity (Fig 6B). In contrast, for the case where growth decreases to very low levels, the reaction norms of Stearns and Koella [3] and our MRN(*r*) specify ever older ages of maturity (Fig 6D).

Finally, note another peculiarity of the fitness function *r*. Because of the particular curvature of the level sets of *r* in age-length space (Fig 3B and 3D), as either linear or von Bertalanffy growth rate decreases, the highest *r* that can be achieved is attained at the same value of maturation length. This extends to unrealistically old ages. This is an unusual feature of attempting to maximize *r* at very low growth rates, especially where *r* < 0.

### Other related work

Our maturation reaction norms describe variation in age (or size) at maturity using accessible data and show how maturation reaction norms respond to variation in growth rate, mortality rate and fecundity. Hutchings and Jones [48] expressed the need for a model similar to ours in their empirical and simulation analyses of Atlantic salmon (*Salmo salar*) maturation reaction norms. We are working to extend our model to iteroparous species, such as Atlantic salmon.

Several investigators have published *empirical* analyses of growth trajectories that influence maturation reaction norms [3,9,25,28,49]. Mangel and Satterthwaite [50] developed a life history model of oceanic salmonids that combines proximate and ultimate mechanisms. They viewed life history as a series of developmental decisions (maturation, smolt) taken at various key times. The outcomes of these decisions depend on whether or not the value of some trait (age or size with an environmental influence) exceeds a (selection determined) threshold. They use *R*_0_ as their underlying fitness function and work around the *R*_0_ = 1 equilibrium.

Lester et al. [25] analyze a two-part growth curve, with linear growth until maturation, and von Bertalanffy growth after maturation. They present an explanation for the parameters of the von Bertalanffy growth curve in terms of pre-maturation growth rate, age at maturation, and annual reproductive investment. They assume that age at maturity and reproductive investment are adjusted to maximize lifetime production of offspring (*R*_0_), accounting for the adult mortality rate. Quince et al. [28,42] extended this analysis [28] and compared it with empirical data [42].

In the study by He and Stewart [9] predictive models of age and size at first reproduction were developed for 85 marine and freshwater fish species (235 populations) based solely on growth parameters. Their study provided predictions of age at first maturity that were consistent with the different life history strategies of fish reported by Winemiller and Rose [51].

### Density dependence

Density dependence can change an individual’s consumption rate, growth rate, fecundity and survival, thus affecting the individual’s age (or size) at maturity and the rate of increase of the individual’s lineage. Ernande et al. [10] looked at the effect of density dependence on fisheries-induced changes in maturation reaction norms. They found that harvesting mature individuals displaces the reaction norm for age and size at maturation and rotates it clockwise, whereas harvesting immature individuals had the reverse qualitative effect. They concluded that the changes in reaction norms were qualitatively similar whether one assumed positive density dependence, no density independence, or negative density dependence.

Mylius and Diekmann [52] compare *r* and *R*_0_ around equilibrium (*r* = 0, *R*_0_ = 1) in a general density dependent system. Their *r* and *R*_0_ depend on type *T* and environment *E*. They find that *R*_0_(*E*, *T*_inv_) leads to an evolutionarily stable strategy (EES) relative to invading types *T*_*i*nv_ if density dependence (invading) reduces life-time offspring production, while *r*(*E*, *T*_inv_) leads to such an ESS if density dependence increases the probability of dying at each age.

Density dependence is not directly addressed in our model. However, changes in density often result in changes in growth rate or mortality rate, especially during the first year of life [35]. The influence of changes in these rates on age and size at maturity can be evaluated in our model.

Size-dependent mortality will be addressed in future versions of this approach.

### Other taxa

Though developed for semelparous fish, our framework is quite general, and could be applied to other taxa. It applies to populations where survivorship to some early size can be estimated, a growth function and constant mortality rate can be specified starting at that age, and fertility can be described as a function of size. We have begun the task of comparing the framework of this paper with observations for multiple fish species.

### Applications for conservation and management

Since different fitness functions lead to different MRNs, using the correct fitness is important for predicting species’ reactions to environmental changes. For example, suppose climate change leads to warming of lake waters, and therefore to faster growth of that lake’s salmon [53]. Conservationists and managers would like to predict the effect on the sizes of individual fish and on the sizes of fish populations. As we have seen, if *r* or *V*_x_ is the underlying fitness function, then as *k* increases, maturation age decreases (while maturation size stays approximately steady). In this case, generation time decreases and the total population increases over the years. On the other hand, if *R*_0_ is the underlying fitness function, the maturation age α –and therefore the larger generation time–remains approximately the same. However, spawning fish will be larger, their fecundity will increase, and, once more, total population will increase. Stream anglers certainly appreciate the increased size of spawning fish–and studies have demonstrated the importance of fish size to generating interest in recreational fishing [54], which in the Great Lakes is worth over $7 billion/yr. The increased individual size that anglers appreciate will only occur if *R*_0_ is the fitness function.

For either fitness function, population increases as *k* increases–because of shorter generation time for *r* or *V*_x_, because of increased fecundity for *R*_0_. A careful geometric analysis of Figs 2 and 3 leads to the conclusion that the population increase is larger when *r* is the fitness function than when *R*_0_ is. Our key point: accurate predictions of population changes resulting from increased *k* require knowing what the underlying fitness function is. (Of course, they also need to take into consideration population-density and size-interaction dependencies.)

Recently, Lake Michigan Chinook Salmon have experienced a slower growth rate because of a dramatic decrease in the Alewife population, their main food source. As Fig 14A indicates, this lowering of *k* has led to a decrease in the size of spawning salmon. It has also led to dramatic decreases in population size.

There are, of course, other semelparous lake fishes for which managers require guidance about maturation age, maturation size, and population size—guidance that requires knowledge of the right fitness function. Consider the Sea Lamprey *Petromyzon marinus*, a parasitic invasive species that invaded the upper Great Lakes in the 1940s and caused extirpation of several stocks of harvested fish [55]. As with Chinook Salmon in the Great Lakes, growth rate and time that Sea Lamprey spend in the lake are dependent on their prey abundance. Control of Sea Lamprey has allowed the valuable sport and commercial fisheries to flourish, and relies on reliable treatment schedules of stream nursery habitats with a pesticide that is toxic to Sea Lamprey. Knowledge of the appropriate fitness is critical for maintaining sustainable fisheries.

Recovery and conservation of declining populations of the American Eel *Anguilla rostrata* also depend on knowing the right fitness function. The American Eel is a semelparous catadromous fish (matures in fresh water, emigrates to spawn in the ocean) whose abundance in the Great Lakes has declined dramatically in the last few decades owing to increased mortality from dams and overharvest [56]. Male eels mature at a fixed length but variable age. By contrast, female eels mature at larger and more variable lengths and fecundities—depending on the latitude, productivity of their juvenile habitat, and mortality risk of a longer spawning migration [57].

Our graphical depiction of reaction norms of changes in age or size at maturity as a function of growth and survival are easily interpretable and useful for managers or conservation planners who wish to understand population consequences of changes in habitat quality or fishing pressure on fish populations.

## Supporting information

S1 Appendix Salmon life history and data sources. (PDF)

S2 Appendix Leslie matrix and definitions of lambda and *r*. (PDF)

S3 Appendix In general, *r* and *V*_x_ yield the same optimal age at maturation. (PDF)

S4 Appendix Optimal length at maturation for maximizing *R*_0_. (PDF)

S5 Appendix Optimal length at maturation for maximizing *r*. (PDF)

S6 Appendix Optimal age and length at maturity to maximize *R*_0_ for von Bertalanffy growth. (PDF)

S7 Appendix Types of responses to slower growth. (PDF)

S1 Dataset Chinook Salmon maturation weight and age in Lake Michigan. (XLSX)

S2 Dataset Chinook Salmon fecundity and weight in Lake Michigan. (CSV)
