# Supplementary material for: The geometry of reaction norms yields insights on classical fitness functions for Great Lakes salmon
Source: PLoS One. 2020 Mar 16;15(3):e0228990. doi: 10.1371/journal.pone.0228990 (PMC7075576; doi:10.1371/journal.pone.0228990)
Supplement: S1 Appendix — (PDF) [file pone.0228990.s001.pdf]

S1 Appendix. Salmon life history and data sources

Salmon Life History

Although our analysis can be applied to many different species, we focus on Great Lakes salmonids because: they were the original source of the questions we consider, some of us have studied them in detail, and their life histories relate in a straightforward way to the model we present.

A number of different salmonids populate the Great Lakes, including Chinook Salmon *Oncorhynchus tshawytscha*, Coho Salmon *O. kisutch*, Pink Salmon *O. gorbuscha*, and Steelhead *O. mykiss*, all of whom originated from Pacific Ocean stock, and Atlantic Salmon *Salmo salar* from Atlantic Ocean stock. The Coho, Pink, and Chinook Salmon are *semelparous*: they put all their energy into laying their first cluster of eggs and die in the process. The Steelhead and Atlantic Salmon are *iteroparous*: they return to lay eggs at their spawning grounds for up to six years before dying from energy losses incurred by spawning.

The Chinook, Coho, Pink, and Atlantic Salmon have similar spawning schedules [1,2]. The spawning females leave the lake and swim up their natal river in the Fall (September-November). In a shallow, fast-flowing segment of the river, the female carves a nest (redd) out of the gravel and lays thousands of eggs. After a male fertilizes the eggs, the female covers them with loose gravel and remains for a few days to guard her nest(s). The semelparous salmon then deteriorate rapidly and die. The iteroparous species swim back to the lake.

The embryos sit in the redd over the winter. Once the weather warms in the spring (March-April), the eggs hatch from the redd. The new salmon (called alevins) stay in the gravel for about a month surviving on the remaining energy in their yolk sacs. When the yolk is gone, the alevins (now called fry) emerge from the gravel redd and grow quickly as they feed on aquatic insects and other benthic invertebrates. As they move down the stream, the salmonid fry acquire vertical brown-green bars (or parr marks) on their sides that provide camouflage and are

called parr. By late summer of their first year (June-July), the Chinook Salmon undergo adaptive physiological, behavioral, color and energetic transformations, are now called smolts, and migrate to sea or to a Great Lake [2].

During the outmigration from their natal tributary to the ocean or Great Lake, salmonid smolts typically undergo heavy predation by birds and fish. In the Great Lakes, the Chinook Salmon smolts are preyed upon by birds, walleye *Sander vitreus*, yearling salmonids, and smallmouth bass *Micropterus dolomieu* [3,4]. Their main predators out in the lake are sea lamprey *Petromyzon marinus* and humans [5,6].

During their first year in the lake, young salmon first feed on insects and zooplankton, then mainly on small preyfish including juvenile alewives and rainbow smelt [7]. After spending 1 to 5 years (typically 2-4 years) in the lake, Chinook Salmon undergo a complex set of physiological processes that enable them to migrate back to their natal freshwater stream to spawn. At this stage they are maturing fish. (Taranger et al. [8] refer to this as attaining puberty.)

Pacific salmonids exhibit quite a bit of variability in the number of years in the river before they smolt. For example, Chinook Salmon have two basic patterns of stream residence [1]. Ocean-type Chinook Salmon migrate downstream after a few months in the river and reside in estuaries for a few weeks or more before moving out to sea or Great Lake. Stream-type Chinook Salmon spend a full year in the river before migrating downstream, exit rapidly from the estuary, and move out to sea. Ocean-type Chinook Salmon return and spawn in fall, while stream-type Chinook Salmon return in spring but spawn in fall. There is also high variability among these four salmonids in time spent in the lake before they first return to the river to spawn. For example, Steelhead reared in the Little Garlic River, a tributary to Lake Superior, may stay three years in the lake before their first spawning run, while Steelhead reared in the Black River and the Little Manistee River in Lake Michigan may wait two years before their first spawning run [9]. This variability in life history traits was a catalyst to the questions we raise in this paper. However, to keep our model tractable, we focus on the Chinook Salmon population of Lake Michigan.

Because of our interest in fertility and age of maturity, we work only with salmonid females.

Chinook Salmon were originally introduced to the Great Lakes in the late 1800s but did not become fully established and naturalized until the late 1960s when they were intensively stocked to feed on an abundant prey source and support a sport fishery [10]. The source of Chinook Salmon stock introduced to Lake Michigan in the 1960s was the Toutle River, Washington population [11].

Data sources

We populated our life history model with data on stream and lake life histories of Lake Michigan Chinook Salmon. We used a variety of sources for these data and compared them with estimates for the same life stages of U.S. and Canadian populations from the northwest Pacific coast. We note where estimates are made directly from field observations or are calculated and provide a qualitative assessment of the estimates.

Stream phase

Survival and fecundity estimates for the stream phase of Chinook Salmon life history came from a mix of empirical and modeling studies conducted on naturalized populations in Lake Michigan and its tributaries, and wild populations in Canadian and US tributaries to the Pacific Ocean (Table A in this Appendix). Salmon abundances at life stages were estimated from studies conducted in the Muskegon River, Michigan, a major producer of naturalized Chinook Salmon smolts in Lake Michigan. O'Neal and Kolb [13] reported the average annual numbers of Chinook Salmon caught from the Muskegon River from 1999-2005 at 17,725, and the number harvested at 5,738.

Table A. Abundances at age or stage, sex ratio or fraction surviving each life stage, and data sources for Chinook Salmon life history in the Muskegon River and Lake Michigan.

Date	Life Stage	Abundance	Fraction Surv.	Data Source and Notes
Oct. 1, prior yr	# Adult spawners	17,215	2/3	(a, b)
Oct. 1, prior yr	# Spawners surviving spawning migration	11,477	0.459	Sex ratio(c)
Oct. 1, prior yr	# Female spawners	5,268	5,485	Fecundity: mean eggs/female(c)
Oct. 1, prior yr	Total pop'n fecundity	28,894,980	0.9025	% Eggs fertilized(d) , predated(e, f)
Oct. 1, Prior yr	# Deposited eggs	26,077,719	0.0207(g)	Egg-fry survival(g)
April 14, yr 0	Total # of fry at hatch	540,000(a)	0.51	Fry-smolt survival(a)
June 14, yr 0	Total wild smolts(b, h)	275,400	0.70	Smolt-lake stage survival(i)
Oct 1, yr 0	# Lake age 0.25	192,780	0.70	(i)
Oct, 1, yr 1	# Lake age 1.25	134,946	0.70	(i)
Oct. 1, yr 2	# Lake age 2.25	94,462	0.70	(i)
Oct. 1, yr 3	# Lake age 3.25	66,123	2/3	(a)
Oct. 1, yr 3	# Spawners	44,082		
Oct. 1, yr 3	# Spawners/female	8.4	0.459	Sex ratio(c)
Oct. 1, yr 3	# Female spawners/female	3.8		

a [12]; b [13]; c [14]; d [1]; e [15]; f [16]; g Estimated as ratio of fry to deposited eggs; h [17]; i [18].

The number of adults spawning in the Muskegon River is unknown. We assumed the numbers of spawners surviving the migration upstream to spawn in the Muskegon River tributary was twice the annual average number of fish harvested (5,738*2 = 11,477). This spawning run size compares with an average spawning run of 15,767 adult Chinook Salmon from 1999-2005 counted at a weir in the Little Manistee River, a Lake Michigan tributary adjacent to Muskegon River, which was supplemented by an average stocking of 508,000 smolts from 1999-2005 [19]. We assumed the average spawning date of Chinook Salmon to be October 1 based on Michigan Dept. Natural Resources creel surveys of Lake Michigan tributary streams from 2000-2005 [3]. Assuming a ratio of 0.459 females to males [14], we estimated the average number of female spawners to be 5,268.

We then estimated total population fecundity from the product of numbers of female spawners and their average fecundity (5,485 eggs, [14]). Fertilization success of Chinook Salmon is rarely measured but assumed to be relatively high, with a median value of 95% [20]. We assumed egg survival from fertilization to deposition to be 95%, as not all fertilized eggs are deposited into the redd owing to predation by resident fishes [15,16] or advection downstream by currents. We have no direct measures of survival from the egg stage to fry emergence from the redd, so we estimated egg-fry survival (S_{egg} = 0.0207) from the ratio of numbers of fry at hatch (see below) to the estimated numbers of eggs deposited. This value of egg survival is lower than reported survival rates [1]. Survival of eggs in the redd is generally assumed to be high [2] but is rarely measured. The few existing estimates of egg survival vary widely ([1], their Table 3, p. 330): Wales and Coots [21] and Coots [22] reported egg survival values varying from 7% to 32% (median = 15%) from egg deposition to the emergent fry stage.

In the Muskegon River, Michigan, the average hatch date of fry from 2005 to 2007 was April 14 [3], calculated from relationships between spawn date, development time and temperature [23]. Average fry abundance at emergence in Muskegon River from 2005-2007 (540,000) was estimated by [12]. Smolt abundance was estimated from 2000-2007 either by electrofishing surveys or by

catch in traps located in the lower Muskegon River, approximately 48 km downstream of spawning areas. The fraction surviving (0.51) from the fry to smolt stage was estimated from the ratio of average smolt to fry abundance from 2005-2007 [12]. The resulting survival rate from egg to smolt stage (l_y) was about 1%. This value is an order of magnitude lower than average egg-smolt survival rates (10%) summarized for Chinook Salmon stocks in western United States and Canada, while our estimate of fry to smolt survival is 3-fold higher than values summarized by Quinn ([3], his Table 15-1). We suspect the discrepancy between egg survival rates may result from uncertainty in actual numbers of eggs deposited that survived in suitable habitats in Muskegon River. Regional differences in survival of fry to the smolt stage may result from lower gradients and flow velocities, and perhaps lower predator densities in Lake Michigan tributaries compared to native tributary habitats in western US and Canada.

Lake phase

We estimated Chinook Salmon survival rates from time of entry into Lake Michigan through lake age 3 from annual estimates of stocking, relative age composition and number returning to weirs during spawning runs, known harvest rates, and natural mortality rates estimated using relationships between fish length, mortality, growth rate characteristics [24]. We estimated survival (s = 0.70) of smolts from the Muskegon River on June 14 to age 0 on Oct 1 in Lake Michigan, and then subsequent annual survival rates of 0.70 [18] from age 0 to age 3, when most salmon spawn [14]. These annual survival rates approximate the annual survival rates of Chinook Salmon estimated from statistical catch at age models run by Michigan State University's Quantitative Fisheries Center for the Lake Michigan Technical Committee Salmonid Working Group (personal communication, R. D. Clark, Jr.). After applying a 67% survival rate during spawning migration, we estimate a ratio of spawners per female of 8.4, which is intermediate to values (6

and 17) calculated by Quinn ([2], his Table 15-1) for Chinook Salmon populations on the US and Canada west coast.

Data on average weight (kg) and length (TL) of Chinook Salmon at maturity, fecundity-weight relationships, number sampled, and sex ratio and alewife biomass were available from for 1991-2013 ([14], their Tables 1 and 2), based on sampling by Michigan Department of Natural Resources at weirs during the fall spawning migration in Lake Michigan. Chinook Salmon fecundity was estimated from the following fecundity-length relationship, based on data described by Kerns et al. [14] (S9 Dataset). The relationship is:

$$\ln(\text{Fecundity}) = -4.192 + 1.891 * \ln(\text{Fish Length, mm}) , \tag{A.1}$$

with N = 172, Adjusted R^2 = 0.29, p < 0.001. Note that the exponent of length (1.891) is noticeably smaller than 3, but is well within the range of values measured for other populations of Chinook Salmon [25]. The von Bertalanffy growth parameters (k = 0.4973 yr^{-1}) and maximum length (L_∞ = 1019 mm TL) were estimated by Wesley [26] from Chinook Salmon length at age data for 1983-1992 and Michigan DNR creel surveys. Natural mortality rate data were obtained from Rutherford [18]. A summary of the parameter values describing the life history of Chinook Salmon in Lake Michigan is presented in Table B. These values are used in the figures.

Table B. Symbols, values, defining equation number, and parameter descriptions for the life history parameters used in the figures (unless otherwise indicated) to represent Chinook Salmon (*Oncorhynchus tshawytscha*) in Lake Michigan. Sources for the values and additional details are given in the text of this appendix.

Parameter	Value	Eq.	Description
A	0.00756	(0.13)	Fertility coefficient of length[a], adjusted for females: 0.5•exp(-4.192)
b	1.891	(0.13)	Fertility exponent of length[a]
C	0.933	(0.33)	$= (1 - L_y / L_\infty)$
k	0.4973 yr^{-1}	(0.33)	von Bertalanffy growth coefficient[b]
l_y	0.00953	(0.33)	Probability at fertilization of surviving to reach Lake Michigan as a smolt
L_y	80 mm	(0.33)	Size upon reaching Lake Michigan as a smolt[c, d]
L_∞	1019 mm	(0.33)	Asymptotic length[b]
q	0.667	(0.12)	Probability of surviving the spawning run up the river to reach the spawning site[e]
s	0.70 yr^{-1}	(0.12)	Probability of surviving one year in Lake Michigan
y	0.701 yr	(0.12)	Age (since fertilization) at reaching Lake Michigan as a smolt
z	0.357 yr^{-1}	(0.12)	$= -\ln(s)$: instantaneous mortality rate

[a]Equation (A.1), (S9 Dataset), [14]; [b] [26]; [c] [27]; [d] [28]; [e] [12]

Salmon-Alewife Interactions

For our discussion in Section 7, we used estimates of population biomass of Chinook Salmon in Lake Michigan by Tsehaye et al. ([6], their Table A3); we used estimates of alewife biomass from USGS-Great Lakes Science Center fall trawl surveys [29]. Chinook Salmon abundances in Lake Michigan increased linearly with stocking levels after the initial introduction in 1967 and increased to a peak in 1985. Salmon abundances then declined in the late 1980s and early 1990s in spite of continued stocking, because of an outbreak of bacterial kidney disease [29]. Salmon abundances increased again through the 1990s after disease-related mortality declined and were relatively constant through the 2000s despite decreases in stocking [30]. The abundance of Alewife, the primary prey of Chinook Salmon in Lake Michigan, fluctuated in inverse proportion to Chinook Salmon biomass through the early 1990s, then declined in 1995 and stayed at low levels through the present [29].

References for Appendix

1. Groot C, Margolis L, editors. Pacific salmon life histories. UBC Press; 1991.
2. Quinn TP. The behavior and ecology of Pacific salmon & trout. Seattle, Washington: University of Washington Press; 2005.
3. Krueger DM, Rutherford ES, Mason DM. Influence of predation mortality on survival of Chinook Salmon parr in a Lake Michigan tributary. Trans. Am. Fish. Soc. 2011; 140: 147-163.
4. Johnson JH, Nack CC, Chalopnicki MA, Abbett R, McKenna Jr JE. Predation on Chinook Salmon parr by hatchery salmonids and Fallfish in the Salmon River, New York. N. Am. J. Fish. Manage. 2016; 36: 74-84.

5. Brenden TO, Bence JR, Szalai EB. An age-structured integrated assessment of Chinook Salmon populations in Lake Huron's main basin since 1968. Trans. Am. Fish. Soc. 2012; 141: 919-933.

6. Tsehaye I, Jones ML, Bence JR, Brenden TO, Madenjian CP, Warner DM. A multispecies statistical age-structured model to assess predator–prey balance: application to an intensively managed Lake Michigan pelagic fish community. Can. J. Fish. Aquat. Sci. 2014; 71: 1-18.

7. Jacobs GR, Madenjian CP, Bunnell DB, Warner DM, Claramunt RM. Chinook Salmon foraging patterns in a changing Lake Michigan. Trans. Am. Fish. Soc. 2013; 142: 362-372.

8. Taranger GL, Carrillo M, Schulz RW, Fontaine P, Zanuy PS, Felip A, Weltzien F-A, Dufour S,. Karlsen Ø, Norberg B, Andersson E, Hansen T. Control of puberty in farmed fish. Gen. Comp. Endocrin. 2010; 165: 483-515.

9. Swank DR. Life -history variation and management of wild Great Lakes Steelhead populations. Ph.D. dissertation, University of Michigan, Ann Arbor. 2005.

10. Carl LM. Natural reproduction of Coho Salmon and Chinook Salmon in some Michigan streams. N. Am. J. Fish. Manage. 1982; 4: 375-380.

11. Borgeson DP, Tody WH, editors. Status report on Great Lakes fisheries. Mich. Dept. Cons. – Fish. Mgmt. Rept. No. 2; 1967.

12. Krueger DM, Rutherford ES, Mason DM. Modeling the influence of parr predation by Walleye and Brown Trout on the long-term population dynamics of Chinook Salmon in Lake Michigan: a stage matrix approach. Trans. Am. Fish. Soc. 2013; 142: 1101-1113.

13. O'Neal RP, Kolb T. Muskegon River Angler Survey Report, 1985 – 2005 Croton Dam to Muskegon Lake, with summaries of lakes, impoundments and other river sections. Michigan Dept. Natural Resources, Fisheries Division Sport Fish Restoration Rept. 230499, 2015.

14. Kerns JA, Rogers MW, Bunnell DB, Claramunt RM, Collingsworth PD. Comparing life history characteristics of Lake Michigan's naturalized and stocked Chinook Salmon. N. Am. J. Fish. Manage. 2016; 36: 1106-1118.

15. Ivan LN, Rutherford ES, Johengen TH. Impacts of adfluvial fish on the ecology of two Great Lakes tributaries. Trans. Am. Fish. Soc. 2011; 140: 1670-1682.

16. Godby Jr NA, Rutherford ES, Mason DM. Diet, consumption, growth, survival, and production of juvenile steelhead in a Lake Michigan tributary. N. Am. J. Fish. Manage. 2007; 27: 578–592.

17. Mason DM, Rutherford ES, Price J. Salmon spawning stock abundance, recruitment and exploitation in the Muskegon River, MI. Project completion report to Great Lakes Fishery Trust for Project 1999.9. 2004; 29 pp.

18. Rutherford ES. Evaluation of natural reproduction, stocking rates, and fishing regulations for steelhead *Oncorhynchus mykiss*, Chinook Salmon *O. tshawytscha*, and Coho Salmon *O. kisutch* in Lake Michigan. Michigan Dept. Natural Resources, Federal Aid in Sport Fish Restoration, Project F-35-R-22, Final Report, Lansing; 1997.

19. Sapak JM, Jonas JL. History of the Little Manistee River harvest weir and fall Chinook Salmon egg-take, 1991-2007. Michigan Dept. Natural Resources, Fish. Tech. Rep.; 2009.

20. Briggs JC. The behavior and reproduction of salmonid fishes in a small coastal stream. Calif. Dep. Fish Game Fish. Bull. 1953; 94: 62 pp.

21. Wales JH, Coots M. Efficiency of Chinook Salmon spawning in Fall Creek, CA. Trans. Am. Fish. Soc. 1954; 84: 137-149.

22. Coots M. The spawning efficiency of King Salmon (*Oncorhynchus tshawytscha*) in Fall Creek, Siskiyou County, California: 1954-55 investigations. Calif. Dep. Fish Game Inland Fish. Admin. Rep. 57-1. 1957, 15 pp.

23. Beacham TD, Murray CB. Temperature, egg size, and development of embryos and alevins of five species of Pacific salmon: a comparative analysis. Trans. Am. Fish. Soc. 1990; 119: 927-945.

24. Pauly D. On the interrelationships between natural mortality, growth parameters, and mean environmental temperature in 175 fish stocks. ICES J. Mar. Sci. 1980; 39: 175-192.

25. Healey MC, Heard WR. Inter- and intra-population variation in the fecundity of Chinook Salmon (*Oncorhynchus tshawytscha*) and its relevance to life history theory. Can. J. Fish. Aquat. Sci. 1984; 41: 476-483.

26. Wesley JK. Age and growth of Chinook Salmon in Lake Michigan: verification, current analysis, and past trends. MS Thesis, University of Michigan, Ann Arbor. 1996; 93 pp.

27. Seelbach PW. Smolt migration of wild and hatchery-raised Coho and Chinook Salmon in a tributary of northern Lake Michigan. Michigan Dept. Natural Resources, Fisheries Res. Rept. 1935. Ann Arbor, MI; 1985.

28. Zafft DJ. Migration of wild Chinook and Coho Salmon smolts from Pere Marquette River, Michigan. MS thesis, Michigan State Univ., East Lansing, MI. 1992.

29. Tsehaye I, Jones ML, Brenden TO, Bence JR, Claramunt RM. Changes in the salmonine community of Lake Michigan and their implications for predator–prey balance. Trans. Am. Fish. Soc. 2014; 143: 420–437.

30. Clark Jr RD, Bence JR, Claramunt RM, Johnson JE, Gonder D, Legler ND, Robillard SR, Dickinson BD. Spatially explicit assessment of changes in Chinook Salmon fisheries in lakes Michigan and Huron from 1986 to 2011. N. Am. J. Fish. Manage. 2016; 36: 1068-1083.
