# Supplementary material for: The geometry of reaction norms yields insights on classical fitness functions for Great Lakes salmon
Source: PLoS One. 2020 Mar 16;15(3):e0228990. doi: 10.1371/journal.pone.0228990 (PMC7075576; doi:10.1371/journal.pone.0228990)
Supplement: S2 Appendix — (PDF) [file pone.0228990.s002.pdf]

S2 Appendix. Leslie Matrix and definitions of lambda and *r*

The basic building blocks of life history theory are x, l_x, and m_x, where x is the age in years since hatching, l_x is the average probability of surviving to age x, and m_x is the average number of offspring of a female of age x. We usually treat age x as a discrete variable in that measurements are taken at discrete times, like the ten-year census of the US population or the passages of salmonids across a weir on their spawning run.

A life history table lists x, l_x, and m_x for each age x. A life history table for a hypothetical iteroparous population that experiences a 50% mortality rate each year and has females that are fertile only in ages 2 and 3 might look like:

x	l_x	m_x
0	1	0
1	.5	0
2	.25	5
3	.125	1
4	0	0

Fishery biologists summarize the complicated life history of salmonids by representing the total age of the fish as a combination of stream age (years spent in the stream before smolting) and lake age (years spent in the lake after smolting). This distinction between stream age and lake age is made because the conditions for growth and survival are usually very different between these environments.

A more dynamic approach to studying life histories uses difference equations and their Leslie Matrix formulation. [See, for example, Caswell (2001)]. Consider a species whose maximum life span is M years, whose females lay their eggs once a year, roughly on the same day, whose eggs hatch soon thereafter, and who are

censused just before they lay their eggs. Let x denote the age in years of an individual starting at hatch ($x = 0$). We call the collection of all (female) individuals with age between x and x+1 "age class x." Let $n_x(t)$ denote the size of age class x at time t. Using life history parameters l_x and m_x, let $s_x = l_{x+1}/l_x$ be the probability of surviving from age x to age x+1. The following system of difference equations keeps track of each age class over time:

$$n_1(t+1) = m_1\, n_1(t) + m_2\, n_2(t) + m_3\, n_3(t) + \ldots. + m_M\, n_M(t)$$
$$n_2(t+1) = s_1\, n_1(t)$$
$$n_3(t+1) = s_2\, n_2(t)$$
$$n_4(t+1) = s_3\, n_3(t), \text{ and so on.}$$

The first equation keeps track of newborns from each age class of females; the rest keep track of survivors from one age class to the next.

In matrix form, this system becomes, for the $M = 4$ case:

$$\begin{pmatrix} n_1(t+1) \\ n_2(t+1) \\ n_3(t+1) \\ n_4(t+1) \end{pmatrix} = \begin{pmatrix} m_1 & m_2 & m_3 & m_4 \\ s_1 & 0 & 0 & 0 \\ 0 & s_2 & 0 & 0 \\ 0 & 0 & s_3 & 0 \end{pmatrix} \begin{pmatrix} n_1(t) \\ n_2(t) \\ n_3(t) \\ n_4(t) \end{pmatrix},$$

or in vector notation $\mathbf{n}(t+1) = \mathbf{A}\,\mathbf{n}(t)$. The matrix $\mathbf{A}$ is called a Leslie matrix. If we iterate this process starting from an initial population distribution $\mathbf{n}(0)$: $\mathbf{n}(1) = \mathbf{A}\,\mathbf{n}(0)$, $\mathbf{n}(2) = \mathbf{A}\,\mathbf{n}(1)$, $\mathbf{n}(3) = \mathbf{A}\,\mathbf{n}(2)$, and so on. Eventually each $\mathbf{n}(t+1)$ will be a constant multiple of the previous $\mathbf{n}(t)$, so that the *relative* size of each age class becomes constant. This multiplier, usually denoted by λ, is called the dominant *eigenvalue* of matrix $\mathbf{A}$. It gives the long run growth rate of the population. The corresponding vector $\mathbf{n}$ of relative sizes of the age classes is called the corresponding eigenvector. To be precise, λ is an eigenvalue for matrix $\mathbf{A}$ and n-tuple $\mathbf{n}$ is a corresponding eigenvector if $\mathbf{A}\,\mathbf{n} = \lambda\,\mathbf{n}$. Any multiple of an eigenvector is

an eigenvector; we often normalize by dividing each entry n_i of **n** by the sum of the n_k*'s* ; in this case we do get an eigenvector whose entries are the long run *proportional size* of each age class. By a bit of algebra (Wikipedia: "Euler-Lotka equation"), one can relate λ to the basic l_x and m_x parameters in a Leslie matrix via Euler's Formula:

$$\frac{m_1\ell_1}{\lambda^1}+\frac{m_2\ell_2}{\lambda^2}+\cdots+\frac{m_T\ell_T}{\lambda^T}=1 \qquad \text{(B.01)}$$

We usually replace the annual growth multiplier λ by its instantaneous counterpart: the so-called "intrinsic growth rate" r, where $e^r = \lambda$ or $r = \ln \lambda$. In this case, Euler's Formula becomes:

$$\frac{m_1\ell_1}{e^{1r}}+\frac{m_2\ell_2}{e^{2r}}+\cdots+\frac{m_T\ell_T}{e^{Tr}}=1. \qquad \text{(B.02)}$$
