# Supplementary material for: The geometry of reaction norms yields insights on classical fitness functions for Great Lakes salmon
Source: PLoS One. 2020 Mar 16;15(3):e0228990. doi: 10.1371/journal.pone.0228990 (PMC7075576; doi:10.1371/journal.pone.0228990)
Supplement: S3 Appendix — (PDF) [file pone.0228990.s003.pdf]

S3 Appendix. In general, *r* and V_x yield the same optimal age at maturation.

We show here that, in general, *r* and each V_x yield the same optimal age at maturation α [17,18]. Using $\lambda^x = e^{rx}$, rewrite the definition (text equation 0.4) of V_x as:

$$\frac{\ell_x V_x}{\lambda^x} = \frac{m_x \ell_x}{\lambda^x} + \frac{m_{x+1}\ell_{x+1}}{\lambda^{x+1}} + \cdots + \frac{m_T \ell_T}{\lambda^{x+T}}. \tag{C.1}$$

The right side of (C.1) has terms for ages *x* to *T*. Substituting the left side of (C.1) for those corresponding terms in Euler's Formula (text equation 0.1) yields:

$$\frac{\ell_1 m_1}{\lambda} + \cdots + \frac{\ell_{x-1} m_{x-1}}{\lambda^{x-1}} + \frac{\ell_x V_x}{\lambda^x} = 1 \tag{C.2}$$

Equation (C.2) is an identity for any λ and V_x. If λ increases in (C.2), V_x must increase too to keep (C.2) an identity. If λ decreases, so must V_x. Of course, λ and $r = \ln \lambda$ always move in the same direction. It follows that any life history trait, including age at maturity, that maximizes *r* must also maximize each V_x [17,18], so that these two fitness functions produce identical values for optimal life history characteristics.
