# Supplementary material for: The geometry of reaction norms yields insights on classical fitness functions for Great Lakes salmon
Source: PLoS One. 2020 Mar 16;15(3):e0228990. doi: 10.1371/journal.pone.0228990 (PMC7075576; doi:10.1371/journal.pone.0228990)
Supplement: S4 Appendix — (PDF) [file pone.0228990.s004.pdf]

S4 Appendix. Optimal length at maturation for maximizing R_0

For maximizing net reproductive rate R_0, use (0.14) and compute the derivative with respect to α. Then find the length that makes the derivative equal to zero.

$$0 = \frac{d}{d\alpha} R_0(\alpha, L(\alpha)) = \frac{d}{d\alpha}\left[c_0 e^{-z\alpha} (L(\alpha))^b \right]$$
$$0 = -z \cdot c_0 e^{-z\alpha} L^b + c_0 e^{-z\alpha} b L^{b-1} L'(\alpha)$$
$$0 = \left(c_0 e^{-z\alpha} L^{b-1} \right)(-zL + bL'(\alpha))$$

where $c_0 = q\ l_y\ A \exp(zy)$.

Then

$$L = \frac{b}{z} L'(\alpha)$$

This general formula can be used with any growth function to determine the optimal length at maturation that maximizes R_0.
