# Supplementary material for: The geometry of reaction norms yields insights on classical fitness functions for Great Lakes salmon
Source: PLoS One. 2020 Mar 16;15(3):e0228990. doi: 10.1371/journal.pone.0228990 (PMC7075576; doi:10.1371/journal.pone.0228990)
Supplement: S5 Appendix — (PDF) [file pone.0228990.s005.pdf]

S5 Appendix. Optimal length at maturation for maximizing *r*

For maximizing population growth rate *r*, use (0.18) and compute the derivative with respect to α. Then find the equation that makes the derivative equal to zero.

$$0 = \frac{d}{d\alpha} r(\alpha, L(\alpha)) = \frac{d}{d\alpha}\left[\frac{1}{\alpha}\left(-b\ln L_{\alpha 0} + b\ln L(\alpha)\right) - z\right]$$
$$0 = -\frac{1}{\alpha^2}\left(-b\ln L_{\alpha 0} + b\ln L(\alpha)\right) + \frac{b}{\alpha}\frac{L'(\alpha)}{L(\alpha)}$$

or

$$L_\alpha[-\ln(L_{\alpha 0}) + \ln(L)] = \alpha L'(\alpha).$$

This general formula can be used with any growth function $L(\alpha)$ to determine the optimal length at maturation that maximizes *r*.
