# Supplementary material for: The geometry of reaction norms yields insights on classical fitness functions for Great Lakes salmon
Source: PLoS One. 2020 Mar 16;15(3):e0228990. doi: 10.1371/journal.pone.0228990 (PMC7075576; doi:10.1371/journal.pone.0228990)
Supplement: S6 Appendix — (PDF) [file pone.0228990.s006.pdf]

S6 Appendix. Optimal age and length at maturity to maximize R_0 for von Bertalanffy growth.

To find the optimal α and L for semelparous species with von Bertalanffy growth using fitness function R_0, we combine the R_0 optimality equation (0.23) and the von Bertalanffy growth equation (0.34) to find:

$$L = \frac{b}{z} L'(\alpha) = \frac{b}{z} \cdot L_\infty C \cdot k \cdot e^{-k(\alpha - y)}. \tag{F.01}$$

Rewrite the first equality in (F.01) as:

$$\frac{d}{d\alpha} \ln L = \frac{L'(\alpha)}{L(\alpha)} = \frac{z}{b} \tag{F.02}$$

or

$$\frac{L_\infty C \cdot k \cdot e^{-k(\alpha-y)}}{L_\infty \left(1 - C \cdot e^{-k(\alpha-y)}\right)} = \frac{z}{b} \text{ or } C \cdot e^{-k(\alpha-y)} = \frac{1}{1 + \frac{bk}{z}} \ . \tag{F.03}$$

Solving (F.03) for α and substituting (F.03) into (0.34), we find that for each growth rate k, the R_0-maximizing age and length at maturity are described by the equations (0.35).
