# Supplementary material for: The geometry of reaction norms yields insights on classical fitness functions for Great Lakes salmon
Source: PLoS One. 2020 Mar 16;15(3):e0228990. doi: 10.1371/journal.pone.0228990 (PMC7075576; doi:10.1371/journal.pone.0228990)
Supplement: S7 Appendix — (PDF) [file pone.0228990.s007.pdf]

S7 Appendix. Types of responses to slower growth.

If fish experience slower growth rates, while other parameters remain the same, then there are nine possible responses, three in terms of age at maturity (age could move earlier, remain the same, or move later) by three in terms of length at maturity (length could become smaller, remain the same, or become larger). Four of these nine combined responses are unlikely: mature at the same age and the same or larger size; or mature at an earlier age and the same or larger size. For example, suppose that under a regime of slower growth, fish matured at the same or larger size. Since it takes more time to reach this size in this new regime, age at maturity automatically increases.

Working only with fitness function *r*, Stearns and Koella ([1]; see also [2]) describe the five remaining patterns they found in their studies of how age and size at maturity respond to decreases in growth rate. "When organisms are forced to grow more slowly, they 1) mature later at a smaller size, 2) mature later at the same size, 3) mature later at a larger size, 4) mature earlier at a smaller size, or 5) mature at the same age at a smaller size" [1, p. 894]. Our maturation reaction norms can describe four of these five patterns, and the remaining pattern could be produced if decreases in growth rate were accompanied by decreases in mortality rate.

We first point out these patterns for fitness function R_0. Three of the patterns (types 1, 4, and 5) are shown in Fig 6 with von Bertalanffy growth. As the growth rate constant *k* in von Bertalanffy growth decreases, the optimal age at maturity moves along MRN(R_0) in a clockwise direction. As growth slows from a fast growth rate at the top of the curve, MRN(R_0) initially predicts responses of their type 1 (mature later at a smaller size), then type 5 (mature at the same age at a smaller size), and lastly type 4 (mature earlier at a smaller size) for the lowest growth rates.

A response of type 5, mature at the same age at a smaller size, is the only pattern in our MRN(R_0) for linear growth, as Fig 2 shows.

A response of type 3, mature later at a larger size, only occurs in our MRN(R_0)s if the reduction in growth occurs with a simultaneous decrease in

mortality rate (*z*) or with an increase in the fecundity exponent of length (*b*).

Turning to fitness function *r*, a response of type 1, mature later at a smaller size, occurs in our MRN(*r*) for von Bertalanffy growth, but only at the highest growth rates (Fig 6C, D; Fig 7).

A response of type 2, mature later at the same size, occurs for our MRN(*r*) with linear growth (Fig 3A, B, C, D; Fig 4) and also in our MRN(*r*) for von Bertalanffy growth at all except the highest growth rates (Fig 6C, D; Fig 7).

This type-2 pattern could also occur for our MRN(R_0) for linear growth (Fig 2) or von Bertalanffy growth (Fig 6A,B) but only if the reduction in growth were matched with a decrease in juvenile mortality rate such that maturation size remained the same. Individuals that were more risk averse might tradeoff a lower growth rate for a lower mortality rate, causing a shift in optimal maturation to a later age but the same size.

Stearns and Koella [1, p. 900] "did not find a combination of parameters or a submodel in which earlier maturity accompanied slower growth." Our MRN(R_0) for von Bertalanffy growth illustrates such earlier maturity as growth rates become very slow (Fig 6A,B). For very slow growth rates, fecundity increases very slowly with age; minor increases in fecundity with age are more than offset by decreases in survival to that age. A decline to very slow growth thus results in decreasing age at first maturity. When growth rates are high, females can attain a substantial fraction of maximal length at a relatively young age (Fig 6). Further increases in growth rate produce only modest increases in fecundity because these fish are already close to their maximum size. But increased growth rate allows high fecundity to be attained in a shorter period of time. An increase in growth rate to very high levels results in a decrease in the optimal age of maturity, because the higher survival (less time for mortality to act) offsets the modest decrease in fecundity due to earlier maturation.

Appendix References

[1] Stearns SC, Koella JC. The evolution of phenotypic plasticity in life-history traits: predictions of reaction norms for age and size at maturity. Evolution. 1986;40: 893-913.

[2] Stearns SC, Crandall RE. Plasticity for age and size at sexual maturity: a life-history response to unavoidable stress. In: Potts GW, Wooton RJ, editors. Fish reproduction: strategies and tactics. London: Academic Press; 1984. pp. 13-33.
